# ﻿A new species of *Saropogon* Loew, 1847 (Diptera, Asilidae) from Arizona, with a review of the Nearctic species north of Mexico

**DOI:** 10.3897/zookeys.1130.81874

**Published:** 2022-11-17

**Authors:** Charlotte H. E. Alberts, Eric M. Fisher

**Affiliations:** 1 Department of Entomology and Nematology, University of California, Davis, USA University of California Davis United States of America; 2 Smithsonian National Museum of Natural History, Washington, D.C., USA Smithsonian National Museum of Natural History Washington United States of America; 3 El Dorado Hills, California, USA unaffiliated El Dorado Hills, California United States of America

**Keywords:** Assassin flies, community science, identification key, Nearctic, robber flies, taxonomy

## Abstract

The Nearctic species of *Saropogon* Loew, 1847 north of Mexico are reviewed, with 19 species recognized and one described as new: *Saropogonpyrodes***sp. nov.** from Arizona. This previously recognized new species has awaited description since its first collection in 1964. Only after a community scientist posted photographs taken in nature to an online database did its description become a priority. All species of *Saropogon* occurring in the Nearctic Region north of the Mexican border have been reexamined. Photographs and diagnoses of all species are provided with a distribution map of the included specimens studied. An updated key to the Nearctic species north of Mexico is provided. Finally, the need for a review of the diverse Mexican fauna is expressed.

## ﻿Introduction

**Figure 1.**
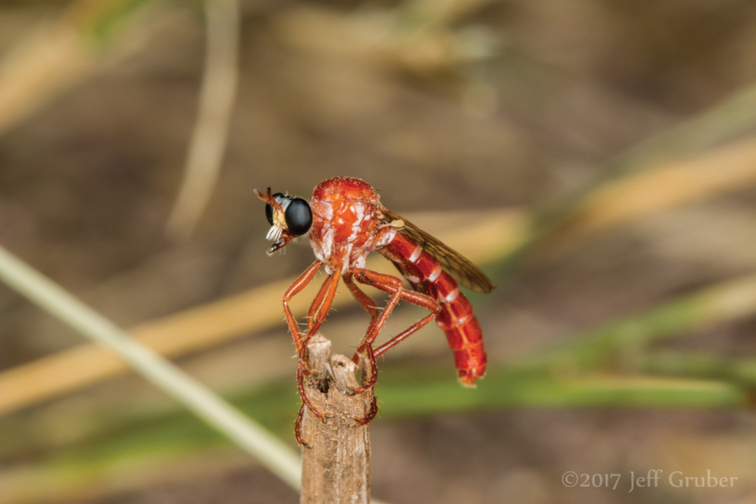
*Saropogonpyrodes* sp. nov. male in nature at ~0.7 km ENE of Amado in southern Arizona on Sep. 5, 2017 (flicker: [https://www.flickr.com/photos/7432824@N07/45297662671/in/album-72157687317436870/]). Photograph by Jeff Gruber.

New and undescribed species of insects are increasingly photographed and posted to online databases by the public (e.g., Mesaglio et al. 2021). Online images and identification databases are excellent resources through which community naturalists and scientists can interact with experts of their interest groups, sometimes resulting in the joint discovery of a new species (e.g., Winterton et al. 2012). Herein we describe a case where a known new species had been awaiting description in a personal collection for many years, but it was not until images were posted online that the naming of the species became a priority. This charismatic and ‘fire-like’ species of assassin fly (Diptera: Asilidae; Fig. 1) has inspired the reexamination of the Nearctic species of the globally diverse and taxonomically confounding genus, *Saropogon* Loew, 1847.

**Figure 2.**
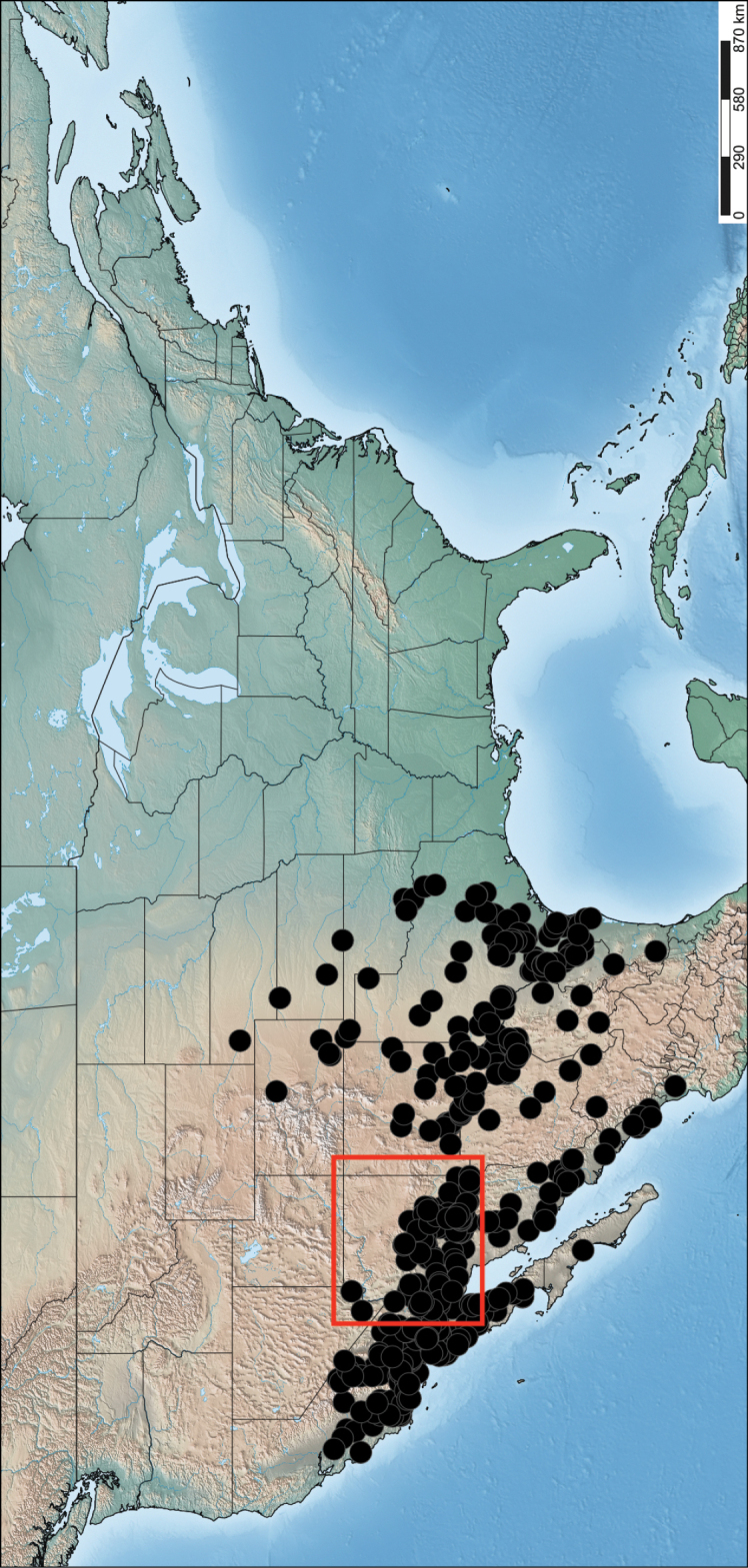
Distribution of Nearctic *Saropogon* (Diptera: Asilidae) examined. Red box indicates focused distribution for Fig. 26. Map created with SimpleMappr on 25 January 2022, and available at: https://www.simplemappr.net/map/17061.

*Saropogon* (Fig. 1) includes at least 128 species and two subspecies (Sakhvon 2020). It is one of few Asilidae genera believed to occur in almost all zoogeographic regions (Londt 1997; Sakhvon 2020; GBIF Secretariat 2021). It is, however, found mainly in temperate and tropical climates. In the Nearctic, *Saropogon* occurs primarily in the southwestern states within the USA, in Texas, Arizona, New Mexico, and California, with some species scattered in the adjacent states. Some species occur as far north as Colorado and Nebraska and as far south as Nayarit, Mexico (Fig. 2). This manuscript focuses on the species found in Arizona but provides locality information of all specimens examined in the Suppl. material 1.

Wilcox (1966) most recently provided descriptions and an identification key to the then known Nearctic species. The status of several species has changed over the years, mainly due to the wide distribution and strong sexual dimorphism of many Nearctic species. We summarize the status history as follows:

- Loew (1847) described *Saropogon* as a subgenus of *Dasypogon* (type species *Dasypogonluctuosus* Wiedemann, 1820).
- Loew (1874) described the first Nearctic *Saropogon* species from Texas (*S.combustus* (male) and *S.adustus* (female)).
- Osten-Sacken (1887) described *Saropogonsenex* from Mexico (Sinaloa).
- Coquillett (1902) described *Saropogondispar* from Texas.
- Johnson (1903) described *Saropogonabbreviates* and *S.bicolor* from Texas.
- Coquillett (1904) described *Saropogonsemiustus*, *S.luteus*, and *S.hyalinus* from California.
- Back (1904) described *Saropogonalbifrons* from Arizona and *S.rufus* from California.
- Back (1909) synonymized *Saropogonalbifrons* with *S.semiustus* (in part, see Wilcox 1966: 131), synonymized *S.adustus* with *S.combustus*, synonymized *S.rufus* with *S.luteus*, and described *S.coquillettii* from New Mexico. He also gave descriptions and a key to the known Nearctic species.
- Curran (1930) described *Saropogonaridus* and *S.purus* from Arizona and published a key to the species.
- Curran (1931) described *Saropogonbirdi* from Oklahoma and provided a revised key to the species.
- Bromley (1934) described *Saropogonfletcheri* and *S.pritchardi* from Texas and Oklahoma and gave a key to the Texas species.
- Wilcox (1936) described the female of *Saropogonaridus*.
- Bromley (1951) described *Saropogonlaparoides* and *S.solus* from Texas.
- Martin and Wilcox (1965) found that *Saropogonaridus* from Arizona was a synonym of *S.senex* described from Sinaloa, Mexico. Included *Saropogonhypomelas* (*Diogmites*) in their catalog.
- Wilcox (1966) described *Saropogonbryanti* and *S.mohawki* from Arizona as well as *S.sculleni* and *S.nitidus* from Texas, noted of the synonymy of *S.albifrons* with *S.semiustus*, and discussed a personal communication with Bromley in 1936, who, after examining the type of *Diogmiteshypomelas* decided that it belonged to *Saropogon* and Wilcox included the change in his identification key.
- Fisher and Wilcox (1997; unpublished) proposed that *Saropogonsculleni* was a junior synonym of *S.laparoides*.

### ﻿Current North American species:

*Saropogonabbreviatus* Johnson, 1903

*Saropogonalbifrons* Back, 1904

*Saropogonbirdi* Curran, 1931

*Saropogonbryanti* Wilcox, 1966

*Saropogoncombustus* Loew, 1874

*Saropogoncoquillettii* Back, 1909

*Saropogondispar* Coquillett, 1902

*Saropogonfletcheri* Bromley, 1934

*Saropogonhyalinus* Coquillett, 1904

*Saropogonhypomelas* Loew, 1866

*Saropogonlaparoides* Bromley, 1951

*Saropogonluteus* Coquillett, 1904

*Saropogonmohawki* Wilcox, 1966

*Saropogonnitidus* Wilcox, 1966

*Saropogonpritchardi* Bromley, 1934

*Saropogonpurus* Curran, 1930

*Saropogonpyrodes* sp. nov.

*Saropogonsemiustus* Coquillett, 1904

*Saropogonsenex* Osten Sacken, 1887

*Saropogonsolus* Bromley, 1951

## ﻿Materials and methods

This study is based on examined specimens from the following institutions and online resources:

**ASUHIC**The Hasbrouck Insect Collection, Arizona State University, Tempe, Arizona, U.S.A.;

**BMEC** The Bohart Museum of Entomology, University of California Davis, Davis, California U.S.A.;

**BugGuide**www.bugguide.net, (VanDyke 2021);

**BYU** Brigham Young University, Provo, Utah, U.S.A.;

**CASENT**California Academy of Sciences Entomology Collection, San Francisco, California U.S.A.;

**Flickr**www.flickr.com;

**iNaturalist**www.inaturalist.org;

**LACMENT**Natural History Museum of Los Angeles County Entomology Collection, Los Angeles, California, U.S.A.;

**MCZ**Museum of Comparative Zoology, Harvard University, Cambridge, Massachusetts, U.S.A.;

**NHMUK**Natural History Museum, London, England, U.K.;

**NMSU** New Mexico State University Arthropod Collection, Las Cruces, New Mexico, U.S.A.;

**TAM** personal collection of Dr. Tristan McKnight, Tucson, Arizona U.S.A.;

**SEMC** Snow Entomological Museum Collection, The University of Kansas, Lawrence, Kansas, U.S.A.;

**TAMUIC**Texas A&M University Insect Collection, College Station, Texas, U.S.A.;

**UAIC**The University of Arizona Insect Collection, Tucson, Arizona, U.S.A.;

**UCR** University of California Riverside Entomology Research Museum, California, U.S.A.; and

**USNM**Smithsonian National Museum of Natural History, Washington, D.C., U.S.A.

Repository abbreviations are from the 2022 GBIF Registry of Scientific Collections with some additions of preferred names from the collection’s website, or personal communications.

Morphological terminology follows Dikow (2009a) and Cumming and Wood (2017). In the descriptions, abdominal tergites are abbreviated with ‘**T**,’ and sternites are abbreviated with ‘**S**.’ Prothoracic, mesothoracic, and metathoracic segments are abbreviated to ‘pro,’ ‘mes,’ and ‘met,’ respectively. Pubescence refers to the short, fine microtrichia densely covering certain body parts. Other generalized terms follow Nichols (1989).

Species descriptions are based on all specimens examined (Suppl. material 1) and not exclusively on the holotype. A total of 1522 specimens of *Saropogon* was examined. The sole specimen of *S.birdi* Curran, 1931 was examined from photographs provided by the AMNH staff. The female wing of *Saropogonpyrodes* was not photographed because only two female specimens were available (the method used is destructive), and because there is no apparent sexual dimorphism present in this species.

Not all holotypes were examined in person. During the research portion of this manuscript, many collections were closed for visits and loans due to the Covid-19 pandemic and specimens were unavailable to the authors. All holotypes were at least examined through photographs. When available, links to all holotype photographs have been provided in the comments section for each species.

In all instances, specimens were dry-mounted on pins. Morphological features were examined using a Wild stereomicroscope. Wing length is measured from the tegula to the distal tip of the wing. Wing length is used in the species descriptions instead of body length because *Saropogon* abdomens are sometimes curved and difficult to measure. We have found more consistent measurements with wing lengths. The left wing was removed or, if previously broken, taken from the unit tray from a representative specimen from each species examined. After being photographed, the wing was then placed in a plastic pill capsule and pinned underneath the relevant specimen. The male terminalia were removed, placed in 10% potassium hydroxide (KOH) at 55 °C, neutralized in acetic acid (CH_3_COOH) and rinsed in distilled water (H_2_O). They were temporarily stored in 75% ethanol (C_2_H_5_OH) for further examination and illustration, eventually sealed in polyethylene vials containing 100% glycerin (C_3_H_8_O_3_), and pinned underneath the corresponding specimen.

Most whole habitus photographs of pinned specimens and wings were taken at the BMEC by the first author, using a GIGAmacro Magnify^2^ system, a Canon MP-E 65 mm macro-lens, Canon EOS Rebel T5i. The specimens were illuminated with a Macro Twin Lite MT-24EX through a simple paper light diffuser tube. The images were then processed through Lightroom and stacked using Zerene stacker. Finally, spot cleaning, color fixing, and inserting scale bars were done in Adobe Photoshop. At USNM, photographs appearing as Fig. 8A–G of the female and male terminalia were taken on a Zeiss SteREO Discovery V12 stereo microscope with a PlanApo S 1.0× lens at 40–95× magnification and an attached Olympus OM-D E-M1 MicroFourThirds digital camera. The dissected terminalia were placed in 75% ethanol in a glass dish and illuminated by a Schott VisiLED light source using mixed bright-field (dorsal), dark-field (lateral), and transillumination (ventral). The MicroFourThirds camera was tethered to a laptop computer and controlled by Olympus Capture software (version 2.2.1), and the vertical movement for obtaining photographs for later image stacking was done manually using the fine drive. Some whole habitus photographs of pinned specimens in the USNM were taken with a GIGAmacro Magnify^2^ system, a Canon EOS D5 Mark IV full-frame DSLR, a Canon MP-E 65 mm F/2.8 macro-lens and illuminated by a Canon ring-lite flash. Individual RAW-format images taken at USNM were stacked using HeliconFocus Pro (version 7+) and exported in Adobe DNG-format.

SimpleMappr was used to generate the distribution maps of all specimens with defined localities (Shorthouse 2010). All localities and elevation not stated explicitly on the original label were estimated using Google Earth Pro version 7.3.4.8248 (Google Earth Pro 2021) and noted as estimates in Suppl. material 1. Google Earth Pro uses digital elevation model (DEM) to calculate elevation.

## ﻿Taxonomy

### ﻿ Saropogon

Taxon classificationAnimaliaDipteraAsilidae

Loew, 1847

B9853132-97C6-5359-ACC8-3A7F9BE90FD0

Saropogon Loew, 1847: 439 (as subgenus of Dasypogon). Type species: Dasypogonluctuosus Wiedemann, 1820; Coquillett (1910: 603); by designation.
= Sarapogon Williston, 1889: 74; incorrect spelling.
= Araiopogon Carrera, 1949: 122; junior synonym. Type species: Dasypogongayi Macquart, 1838: 37).
= Lycomax Hull, 1962: 278; as a subgenus of Saropogon Loew, 1847. Type species: Saropogonflavofacialis Hull, 1956: 133.
= Oberon Carrera & Papavero, 1962: 57; junior synonym. Type species: Oberonvelutinus Carrera & Papavero, 1962: 58.

#### Subfamily.

Dasypogoninae (Hull 1962; Papavero 1973; Artigas and Papavero 1988; Lehr 1988; Geller-Grimm 2004; Dikow 2009a; Cohen et al. 2021).

#### Tribe.

Saropogonini (Hardy 1926; Martin and Papavero 1970; Dikow 2009a, 2009b, 2018).

#### Diagnosis.

**Figure 3.**
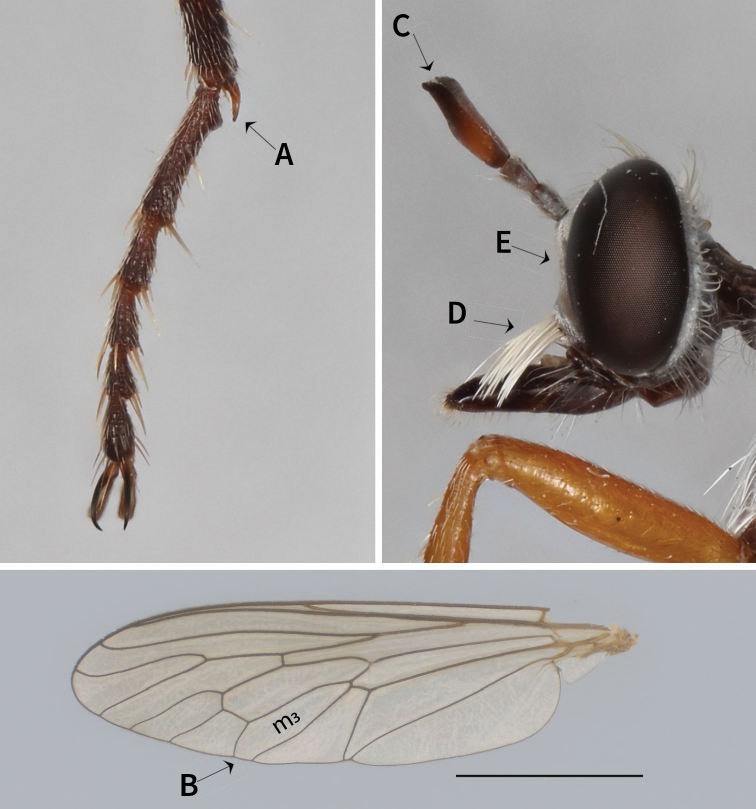
*Saropogonnitidus* illustrating distinguishing characters of the genus **A** fore tibia with a distinct spur. **B** open m_3_ cell on wing **C** antennal style **D** mystax of *S.nitidus* restricted to oral margin **E** face slightly concave. Scale bar: 2 mm.

*Saropogon* has a stout and often twisted spur at the antero-ventral apex of the fore tibiae (Fig. 3A), the same as related genera in the subfamily Dasypogoninae. It differs from other Nearctic taxa such as *Diogmites* Loew and *Blepharepium* Rondani by having cell m_3_ open (Fig. 3B), and an antennal stylus composed of a single element with an apical seta-like element positioned apically in a cavity on the stylus (Fig. 3C). However, some *S.pritchardi* have cell m_3_ almost closed, but never stalked. *Saropogon* differs from *Lestomyia* Williston by having a mystax confined to the oral margin (Fig. 3D) and its face is slightly concave (Fig. 3E) when viewed laterally. Some species of *Lestomyia* have a mystax confined to the oral margin, which can be distinguished from *Saropogon* by having strong anterior (presutural) dorsocentral bristles (absent in *Saropogon* (Wilcox 1966)). *Cophura* can be distinguished from *Saropogon* by its fore tibial spur on the postero-ventral surface being thin, and sigmoid rather than stout, hooked and on the antero-ventral surface (Dikow 2009a). *Cophura* also has a midtibia with a large, usually black, apical spine, which is absent in all *Saropogon* studied. Length 10–27 mm.

**Figure 4.**
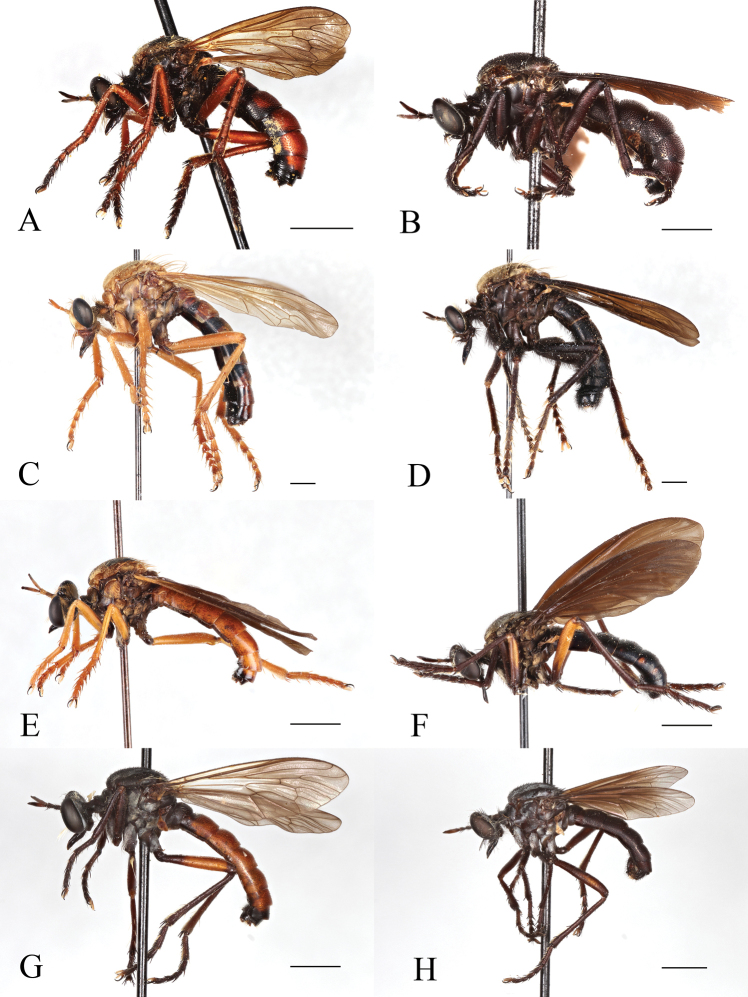
Sexual color dimorphism **A***Saropogonabbreviatus* female **B***S.abbreviatus* male **C***S.combustus* female **D***S.combustus* male **E***S.purus* female **F***S.purus* male **G***S.senex* female **H***S.senex* male. Scale bars: 2 mm.

Sexual Dimorphism and wing variation in *Saropogon*. Back (1909) and Wilcox (1966) have called attention to many species of *Saropogon* that represent prime examples of sexual dimorphism. Species like *S.abbreviatus* (Fig. 4A, B), *S.combustus* (Fig. 4C, D), *S.purus* (Fig. 4E, F), and *S.senex* (Fig. 4G, H) have the male abdomen predominantly black, whereas the female abdomen is largely red. However, there can be color variation within these species. Curran (1931) reported a female *S.combustus* with a black abdomen. Leg color is also sexually dimorphic in most Nearctic *Saropogon*, with male legs tending to be black and female legs mainly reddish. Exceptions occur: the male of *Saropogonpurus* has reddish hind femora and middle femora, and the female of *S.senex* has mainly black legs except for reddish hind femora. Setal patterns can also be dimorphic: males have long, erect, or semierect hairs on the mesonotum, abdomen, and legs in *Saropogonbryanti*, *S.combustus*, *S.coquillettii*, *S.dispar*, *S.laparoides*, and *S.mohawki*. In the females of these species, these hairs are short, appressed, and inconspicuous.

**Figure 5.**
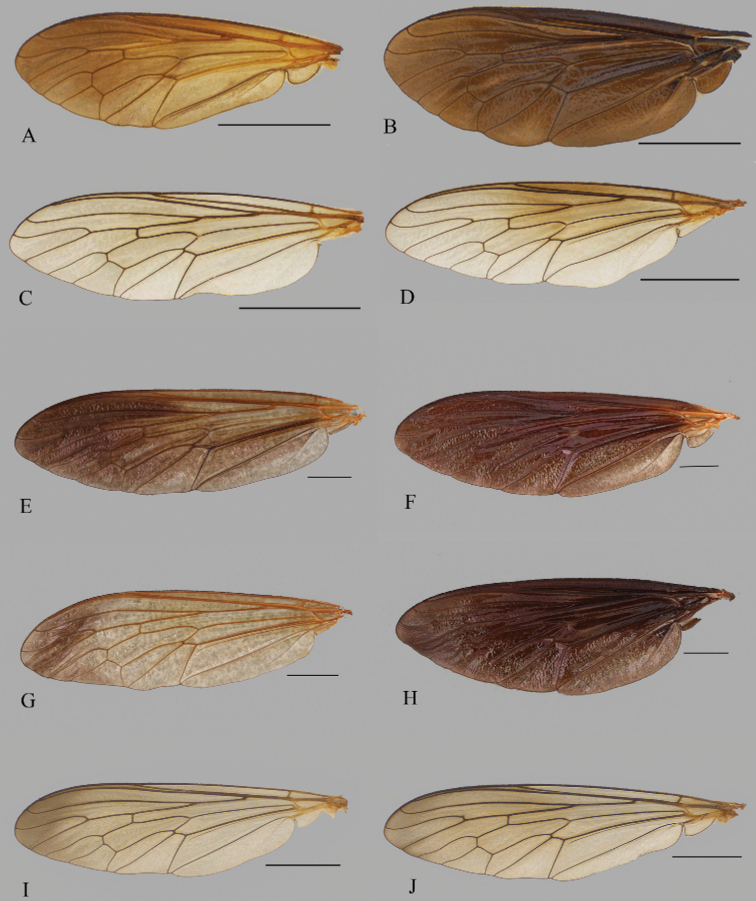
Representative *Saropogon* wings of **A***S.abbreviatus* female **B***S.abbreviatus* male **C***S.bryanti* female **D***S.bryanti* male **E***S.combustus* female **F***S.combustus* male **G***S.dispar* female **H***S.dispar* male **I***S.hypomelas* female **J***S.hypomelas* male **K***S.luteus* female **L***S.luteus* male **M***S.purus* female **N***S.purus* male, and **O***S.senex* female **P***S.senex* male. Scale bars: 2 mm.

Wilcox (1966) emphasized that the wings of many species of *Saropogon* contain diagnostic features. Wings of *Saropogonabbreviatus* (Fig. 5A, B), *S.bryanti* (Fig. 5C, D), *S.combustus* (Fig. 5E, F), *S.dispar* (Fig. 5G, H), *S.hypomelas* (Fig. 5I, J), *S.luteus* (Fig. 5K, L), *S.purus* (Fig. 5M, N), and *S.senex* (Fig. 5O, P) are sexually dimorphic: they are brown in males, yellowish in females. Species with brown wings in both sexes are *Saropogonsenex*, *S.abbreviatus*, *S.purus*, and *S.pritchardi*; *S.luteus* and *S.pyrodes* sp. nov., have yellowish wings in both sexes.

#### Biology.

Dasypogoninae and *Saropogon* apparently tend to prefer Hymenoptera prey (Lavigne 2016; Pollock 2021; Table 1). *S.combustus* and *S.pritchardi* show a particular interest in the workers of *Pogonomyrmex* harvester ants (Pollock 2021). There is currently only one record of *Saropogon* as prey to another genus of Asilidae in North America. Bromley (1934) recorded *Diogmitessymmachus* Loew, 1872 feeding on *Saropogondispar* in Texas.

**Table 1.** Adult *Saropogon* predation records in North America. Records gathered from Lavigne 2016 online database (specimens were not examined personally); Arizona State University, Hasbrouck Insect Collection (ASUHIC); Bellamy 2002; Brigham Young University, Provo, Utah (BYU); University of California, Davis, The Bohart Museum of Entomology (BMEC); Bromley 1934; Hurd 1952; Hurd and Linsley 1975; New Mexico State University Arthropod collection (NMSU); Pollock 2021; Sweetman 1958; Texas A&M University insect collection (TAMUIC); Thorp 1973; University of Arizona Insect Collection (UAIC); University of California, Riverside, Entomology Research Collection (UCR), and the Smithsonian’s National Museum of Natural History (USNM) pinned collection. Duplicate prey records for the same species are not included.

Predator	Prey order	Prey family	Original source or collection	Country (state)
*S.abbreviatus*	Hymenoptera	Apidae	BYU	USA (TX)
*S.albifrons*	Hymenoptera	Crabronidae	UCR	USA (CA)
*S.bryanti*	Hymenoptera	Apidae	USNM	USA (AZ)
*S.bryanti*	Hymenoptera	Vespidae	UAIC	USA (AZ)
*S.bryanti*	Hymenoptera	(?)	ASUHIC	USA (AZ)
*S.combustus*	Coleoptera	Carabidae	Pollock 2021	USA (NM)
*S.combustus*	Coleoptera	Chrysomelidae	Pollock 2021	USA (NM)
*S.combustus*	Coleoptera	Tenebrionidae	Pollock 2021	USA (NM)
*S.combustus*	Diptera	Asilidae	Pollock 2021	USA (NM)
*S.combustus*	Diptera	Bombyliidae	Pollock 2021	USA (NM)
*S.combustus*	Diptera	Culicidae	Pollock 2021	USA (NM)
*S.combustus*	Hemiptera	Cicadidae	Pollock 2021	USA (NM)
*S.combustus*	Hemiptera	Membracidae	Pollock 2021	USA (NM)
*S.combustus*	Hemiptera	Rhopalidae	Pollock 2021	USA (NM)
*S.combustus*	Hymenoptera	Andrenidae	Pollock 2021	USA (NM)
*S.combustus*	Hymenoptera	Apidae	Pollock 2021	USA (NM)
*S.combustus*	Hymenoptera	Apoidea	Pollock 2021	USA (NM)
*S.combustus*	Hymenoptera	Braconidae	Pollock 2021	USA (NM)
*S.combustus*	Hymenoptera	Crabronidae	Pollock 2021	USA (NM)
*S.combustus*	Hymenoptera	Formicidae	Pollock 2021	USA (NM)
*S.combustus*	Hymenoptera	Formicidae	Pollock 2021	USA (TX)
*S.combustus*	Hymenoptera	Halictidae	NMSU	USA (NM)
*S.combustus*	Hymenoptera	Ichneumonidae	Pollock 2021	USA (NM)
*S.combustus*	Hymenoptera	Mutillidae	Pollock 2021	USA (NM)
*S.combustus*	Hymenoptera	Pompilidae	Pollock 2021	USA (NM)
*S.combustus*	Hymenoptera	Sphecidae	Pollock 2021	USA (NM)
*S.combustus*	Hymenoptera	Thynnidae	Pollock 2021	USA (NM)
*S.combustus*	Hymenoptera	Tiphiidae	Pollock 2021	USA (NM)
*S.combustus*	Hymenoptera	Vespidae	Pollock 2021	USA (NM)
*S.combustus*	Araneae	(?)	Pollock 2021	USA (NM)
*S.coquillettii*	Hymenoptera	Apidae	TAMUIC	USA (TX)
*S.coquillettii*	Hymenoptera	Apidae	Hurd and Linsley 1975	USA (NM)
*S.coquillettii*	Hymenoptera	Megachilidae	Hurd and Linsley 1975	USA (NM)
*S.coquillettii*	Hymenoptera	Vespidae	NMSU	USA (NM)
*S.dispar*	Coleoptera	Cerambycidae	USNM	USA (TX)
*S.dispar*	Coleoptera	Elateridae	Sweetman 1958	USA (?)
*S.dispar*	Coleoptera	Scarabaeidae	Sweetman 1958	USA (?)
*S.dispar*	Diptera	Bombyliidae	TAMUIC	USA (TX)
*S.dispar*	Diptera	Bombyliidae	Bromley 1934	USA (TX)
*S.dispar*	Diptera	Calliphoridae	USNM	USA (TX)
*S.dispar*	Diptera	Muscidae	TAMUIC	USA (TX)
*S.dispar*	Diptera	Syrphidae	Bromley 1934	USA (TX)
*S.dispar*	Hemiptera	Coreidae	Bromley 1934	USA (TX)
*S.dispar*	Hymenoptera	Andrenidae	Bromley 1934	USA (TX)
*S.dispar*	Hymenoptera	Apidae	BMEC and Thorp 1973	USA (OK)
*S.dispar*	Hymenoptera	Apidae	USNM, BYU	USA (TX)
*S.dispar*	Hymenoptera	Crabronidae	BMEC	USA (OK)
*S.dispar*	Hymenoptera	Halictidae	Bromley 1934	USA (TX)
*S.dispar*	Hymenoptera	Halictidae	Thorp 1973	USA (OK)
*S.dispar*	Hymenoptera	Pompilidae	TAMUIC	USA (TX)
*S.dispar*	Hymenoptera	Scoliidae	Bromley 1934	USA (TX)
*S.dispar*	Hymenoptera	Sphecidae	Bromley 1934	USA (TX)
*S.dispar*	Hymenoptera	Sphecidae	BMEC and Thorp 1973	USA (OK)
*S.dispar*	Hymenoptera	Vespidae	Bromley 1934	USA (TX)
*S.dispar*	Orthoptera	Acrididae	Bromley 1934	USA (TX)
*S.fletcheri*	Coleoptera	Buprestidae	BYU	USA (TX)
*S.fletcheri*	Hymenoptera	Scoliidae	BYU	USA (TX)
*S.fletcheri*	Hymenoptera	Vespidae	BYU	USA (TX)
*S.fletcheri*	Hymenoptera	(?)	BYU	USA (TX)
*S.hypomelas*	Hymenoptera	Ichneumonidae	TAMUIC	USA (TX)
*S.hypomelas*	Hymenoptera	Vespidae	TAMUIC, USNM	USA (TX)
*S.mohawki*	Coleoptera	Buprestidae	Bellamy 2002, USNM	USA (CA)
*S.mohawki*	Hymenoptera	Halictidae	USNM	MEX (B.C.N.)
*S.mohawki*	Hymenoptera	(?)	ASUHIC	USA (AZ)
*S.pritchardi*	Coleoptera	Carabidae	Pollock 2021	USA (NM)
*S.pritchardi*	Coleoptera	Tenebrionidae	Pollock 2021	USA (NM)
*S.pritchardi*	Hymenoptera	Formicidae	Pollock 2021	USA (NM)
*S.pritchardi*	Hymenoptera	Formicidae	Pollock 2021	USA (TX)
*S.purus*	Diptera	(?)	ASUHIC	USA (AZ)
*S.purus*	Hymenoptera	(?)	ASUHIC	USA (AZ)
*S.pyrodes*	Hymenoptera	Apidae	Photograph – Jeff Gruber	USA (AZ)
*S.senex*	Coleoptera	Elateridae	USNM	MEX (Nay)
*S.senex*	Hymenoptera	Formicidae	USNM	MEX (Nay)

*Saropogon* females oviposit in soil with the aid of the acanthophorite spines (Fig. 25D) at the tip of their ovipositor (Londt and Dikow 2017). They use the spines to dig into the ground, to lay the eggs, and to sweep soil over the eggs after oviposition (Dennis and Lavigne 1975).

### ﻿Key to species of North American *Saropogon*, modified from Wilcox (1966)

**Table:** 

1	Apical scutellar macrosetae absent or short, shorter than ½ length of scutellum	**2**
–	Apical scutellar macrosetae present, as long or longer than length of scutellum	**4**
2	Apical scutellar macrosetae absent; both sexes with reddish abdomen; wing length 8 mm (USA: Texas; Mexico: Tamaulipas) Fig. 30	***S.solus* Bromley**
–	Apical scutellar macrosetae present; male abdomen black, female abdomen reddish	**3**
3	Discal scutellar setae developed as short macrosetae; anepisternum (except dorsally), katepisternum, proepimeron, and anepimeron non-pubescent with large, uniformly arranged circular depressions; male legs black, female legs red (USA: California, Texas; Mexico: Baja California, Tamaulipas) Fig. 6	***S.abbreviatus* Johnson**
–	Discal scutellar setae absent; anepisternum, katepisternum, proepimeron, and anepimeron with grayish pubescence, without uniformly arranged circular depressions; legs predominantly black, both sexes with metathoracic femora red (USA: Arizona; Mexico: Sinaloa, Sonora, Nayarit) Fig. 29	***S.senex* Osten Sacken**
4	Wings hyaline, without microtrichia or sparse microtrichia apically with no or sometimes slight color staining	**5**
–	Wings infuscate, males with brown or black wings, females paler but with staining and/or microtrichia concentrated apically and around veins; generally larger flies (except *S.purus* and *S.luteus*)	**12**
5	Predominantly black abdomen; fore coxae with long, fine, white setae (USA: Texas) Fig. 16	***S.laparoides* Bromley**
–	Predominantly reddish or yellowish abdomen; fore coxae with macrosetae or bare	**6**
6	Anepisternum and katepisternum with non-pubescent spot on the anterior half (e.g., Fig. 19B, F)	**7**
–	Anepisternum and katepisternum pubescent throughout	**8**
7	Red non-pubescent spot on anepisternum and katepisternum; femora reddish; antennae dark red to yellow; wings with slight microtrichia apically (USA: Arizona) Figs 22, 23–27	***S.pyrodes* sp. nov.**
–	Black non-pubescent spot on anepisternum and katepisternum; femora yellowish; antennae black to brown; wings entirely bare of microtrichia (USA: New Mexico, Texas; Mexico: Chihuahua, Coahuila) Fig. 19	***S.nitidus* Wilcox**
8	White macrosetae on scutum and scutellum; scutellum with gray pubescence	**9**
–	Yellowish macrosetae on scutum and scutellum; scutellum with gold pubescence	**10**
9	Face and anepisternum with pale gold pubescence; male legs black with distally red femora, female with reddish legs; wings completely hyaline (USA: California, Arizona; Mexico) Fig. 28	***S.semiustus* Coquillett**
–	Face and anepisternum with gray pubescence; both sexes with reddish legs; wings mostly hyaline but with slight brown tinge anteroproximally (USA: Arizona, California; Mexico: Baja California) Fig. 7	***S.albifrons* Back**
10	Wings mostly hyaline but always with slight microtrichia apically; male femora proximally black over half the length, females with entirely reddish legs (USA: Arizona, New Mexico, Texas; Mexico: Sonora) Fig. 11	***S.coquillettii* Back**
–	Wings completely hyaline; both sexes with reddish legs, sometimes femora proximally darker but never more than half the length	**11**
11	Abdomen T4 and 5 anterolaterally black in both sexes; four apical scutellar macrosetae; male femora sometimes proximally black and reddish distally, female legs entirely reddish (USA: Arizona, California, Nevada, Utah; Mexico: Baja California, Sonora) Fig. 18	***S.mohawki* Wilcox**
–	Abdomen yellow; two apical scutellar macrosetae; both sexes have entirely reddish legs (USA: New Mexico, Texas; Mexico: Chihuahua, Coahuila) Fig. 14	***S.hyalinus* Coquillett**
12	Small flies (body length < 15 mm; wing length < 11 mm)	**13**
–	Large flies (body length > 15 mm; wing length > 11 mm)	**14**
13	Wings pale orange stained especially around veins, microtrichia apically, thin (width < 1/3 of length); both sexes with thorax and abdomen orange (USA: California; Mexico: Baja California) Fig. 17	***S.luteus* Coquillett**
–	Wings entirely dark brown from microtrichia and wide (width > 1/3 of length); male with black thorax and abdomen, female with dark brown thorax and orange abdomen (USA: Arizona; Mexico: Sinaloa, Sonora) Fig. 21	***S.purus* Curran**
14	Femora entirely red (e.g., Fig. 13B)	**15**
–	Femora entirely black or at least with a dorsal black stripe (e.g., Fig. 8B, C)	**17**
15	T2–4 non-pubescent to sparse white pubescence on posterolateral margin, narrowly black on the anterior margins forming a thin band (USA: Texas) Fig. 13	***S.fletcheri* Bromley**
–	T2–4 white pubescence on posterolateral margin, if black on the anterior margin, never forming a thin band	**16**
16	Wings entirely dark brown from microtrichia; antennae brown (USA: New Mexico, Oklahoma, Texas) Fig. 20	***S.pritchardi* Bromley**
–	Wings pale orange stained especially around veins, microtrichia apically; antennae orange (USA: Colorado, Kansas, Nebraska, New Mexico, Oklahoma, Texas) Fig. 4, 10	***S.combustus* Loew - in part (females)**
17	Coxae and katatergite with black setae (USA: Colorado, Kansas, Nebraska, New Mexico, Oklahoma, Texas) Figs 4, 10	***S.combustus* Loew - in part (males)**
–	Coxae and katatergite with white or yellow setae	**18**
18	Abdomen predominantly black; T3 red is restricted to the posterior half if any	**19**
–	Abdomen predominantly red; T3 black is restricted to the antero-lateral surface	**20**
19	Female with black basal segments of the palpi, segment 2 reddish; abdomen mostly black; two apical scutellar macrosetae (USA: Oklahoma) Fig. 8	***S.birdi* Curran –(females)**
–	Female with orange basal segments of the palpi, male with black; female abdomen with some black; male abdomen mostly black; four apical scutellar macrosetae (USA: Oklahoma, Texas) Fig. 12	***S.dispar* Coquillett**
20	Male face and frons with white pubescence, female golden with ocellar tubercle and area around it white; male femur, sometimes tibia, black; female femur proximally black or with proximal black dorsal stripe, legs reddish; scutum with yellowish gray pubescence median stripe with brown pubescence without sub-lateral spots (USA: Arizona, New Mexico, Texas; Mexico: Coahuila, Nuevo Leon) Fig. 15	***S.hypomelas* (Loew)**
–	Both sexes face and frons with golden pubescence; femur in both sexes reddish with black dorsal stripe; scutum yellowish with broad central stripe and elongated sub-lateral spots with gray pubescence (USA: Arizona, New Mexico, Texas; Mexico: Sonora) Fig. 9	***S.bryanti* Wilcox**

### ﻿ Saropogon abbreviatus

Taxon classificationAnimaliaDipteraAsilidae

Johnson, 1903

611412D7-17D5-5C3F-B94F-66565F871A87

**Figure 6.**
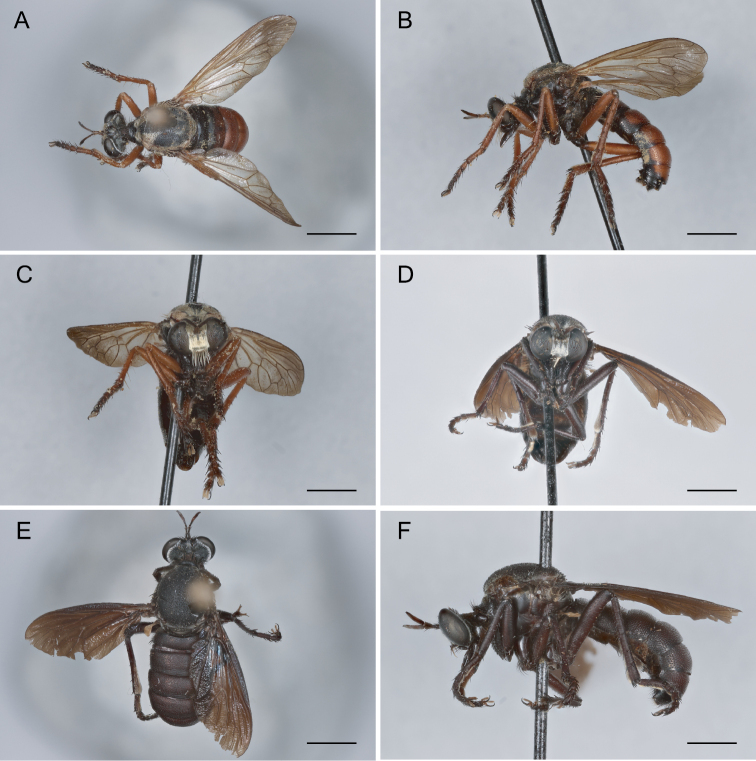
*Saropogonabbreviatus* Johnson, 1903 Female (USNMENT01830071): **A** dorsal view **B** lateral view **C** anterior view; Male (USNMENT01830070): **D** anterior view **E** dorsal view **F** lateral view. Scale bars: 2 mm.

Figs 4A, B , 5A, B , 6 , 26 , 31

Saropogon abbreviatus Johnson, 1903: 113.
Saropogon bicolor Johnson, 1903: 113, junior synonym [homonym of Saropogonbicolor Jaennicke, 1867 (currently recognized as Diogmitesbicolor Jaennicke, 1867)].

#### References.

Back 1909: 345 (key and redescription); Curran 1930: 2 (key), 1931: 2 (key); Martin and Wilcox 1965: 383 (catalog); Wilcox 1966: 128 (key); Fisher and Wilcox 1997: 4 (catalog).

#### Diagnosis.

Has a rather short and stout abdomen with uniformly arranged circular depressions. The male is black with black or brown wings and the female is reddish with brown wings, darker apically. Body length 9–12 mm; wing length 7–9 mm. Flight time April – August.

Most similar to *Saropogonsenex* and *S.purus*. Differs from *S.purus* because *S.abbreviatus* has short apical scutellar macrosetae, whereas the apical scutellar macrosetae of *S.purus* are longer than the length of the scutellum. Differs from *S.senex* because *S.abbreviatus* has short discal scutellar macrosetae, and *S.senex* has none.

#### Distribution.

USA: California, Texas; Mexico: Baja California, Tamaulipas.

#### Type material examined.

United States of America • 1 ♂, holotype; Texas; MCZ; Type 7582.

#### Other material examined.

Suppl. material 1.

#### Comments.

The holotypes of *Saropogonabbreviatus* and *S.bicolor* (jr. syn.) are currently in the Museum of Comparative Zoology at Harvard University. The collection provides photos of the types on their website MCZBase: MCZ:Ent:7582 https://mczbase.mcz.harvard.edu/guid/MCZ:Ent:7582 and MCZ:Ent:32756 https://mczbase.mcz.harvard.edu/guid/MCZ:Ent:32756.

### ﻿ Saropogon albifrons

Taxon classificationAnimaliaDipteraAsilidae

Back, 1904

90D62D86-52B7-5D2D-B2A0-E1246653D329

Figs 7 , 26 , 32

Saropogon albifrons Back, 1904: 29.
Saropogon semiustus Coquillett, 1904: 186, junior synonym. In part.

#### References.

Martin and Wilcox 1965: 383 (catalog); Wilcox 1966: 130 (key and redescription); Fisher and Wilcox 1997: 4 (catalog).

**Figure 7.**
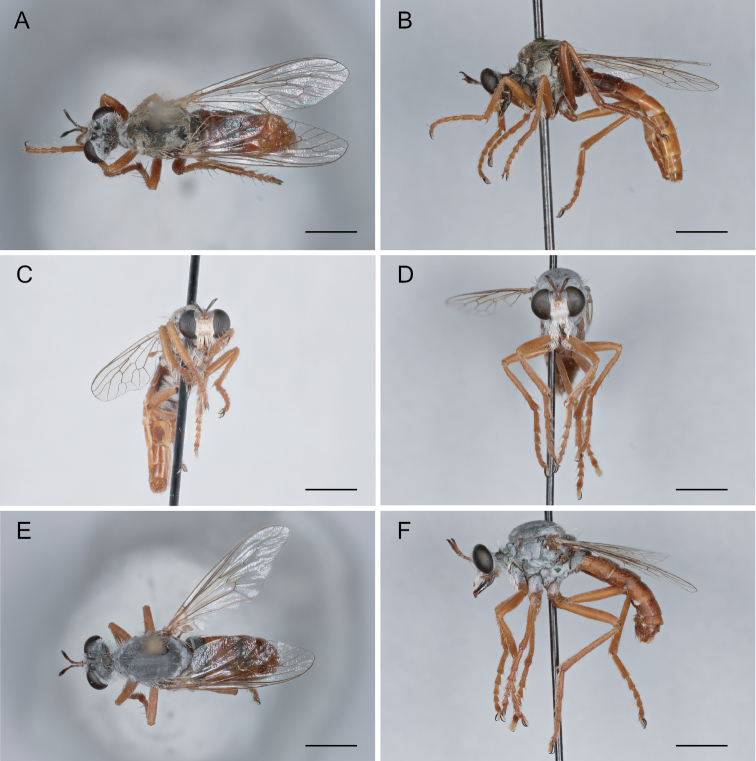
*Saropogonalbifrons* Back, 1904 Female (USNMENT01819164): **A** dorsal view **B** lateral view **C** anterior view; Male (USNMENT01830072): **D** anterior view **E** dorsal view **F** lateral view. Scale bars: 2 mm.

#### Diagnosis.

Legs reddish orange in both sexes; face, scutum, and anepisternum entirely with white pubescence with white macrosetae; antennae yellowish; ~ 30 macrosetae forming mystax; wings hyaline with a slightly darker tinge proximally; veins brownish at the base of the wing, darker apically; T2–5 postero-laterally with white pubescence in both sexes; scutellum with only two marginal bristles. Body length 9–14 mm; wing length 7–9 mm. Flight time April – June.

Easily confused with *Saropogonsemiustus*, especially females; white face pubescence is the best distinguishing character in *S.albifrons*.

#### Distribution.

USA: Arizona, California; Mexico: Baja California.

#### Type material examined.

**United States of America** • 1 ♀, lectotype; Arizona, Mohave County, Bill Williams Fork; August; F. H. Snow; SEMC; SEMC1603972 • 1 ♀, paralectotype; same collection information as lectotype; SEMC; SEMC1603973.

#### Arizona material examined.

United States of America • 6 ♀; La Paz County, Parker, Osborn Well Road, 1.6 km E. of Route 95, white sand dunes; 34°07'N, 114°15'W; 150 m; 02 May 2008; T. Dikow, E. Fisher; USNM; USNMENT00870564, USNMENT00870565, USNMENT00870566, USNMENT00870567, USNMENT00870568, USNMENT00870569 • 1 ?; Maricopa County, Bush Highway; 33°32'N, 111°35'W; 415 m; 09 May 1968; R. N. Foster; ASUHIC; ASUHIC0139490 • 1 ♀; Maricopa County, Gila Bend; 32°56'N, 112°43'W; 224 m; F. H. Parker; USNM; USNMENT0119937 • 3♂, 1♀; Maricopa County; Gila River, 10 km S. Arlington; 33°13'N, 112°45'W; 200 m; 03 June 2010; F. D. Parker, M. E. Irwin; UAIC • 1 ♀; Maricopa County; Queen Creek; 33°15'N, 111°38'17"W; 425 m; 06 June 1964; G. D. Butler Jr.; UAIC • 1 ?; Yuma County; 8 mi. SE of Parker; 34°01'N, 114°01'W; 176 m; 07 May 1966; S. A. Gorodenski, J. M. Davidson, M. A. Cazier; ASUHIC; ASUHIC0139489 • 1 ?; Yuma County, Mohawk Pass; 32°43'N, 113°44'W; 24 April, 1966; J. H. Davidson, J. M. Davidson, M. A. Cazier; ASUHIC; ASUHIC0139488.

#### Other material examined.

Suppl. material 1.

#### Comments.

*Saropogonalbifrons* was not mentioned by Curran (1930, 1931), most likely because the species was not included in the Back (1909) identification key. [The authors are unsure as to why it was not included.] The co-types (syntypes) referenced in Back 1904 were deposited one in the Massachusetts Agricultural College collection and one at the University of Kansas collection (SEMC); however, both can be currently found at SEMC. The authors have designated the specimen in better condition to be the lectotype and the other the paralectotype. Information about them can be found here: https://biodiversity.ku.edu/node/1095/.

### ﻿ Saropogon birdi

Taxon classificationAnimaliaDipteraAsilidae

Curran, 1931

73C82FC0-FF55-51F4-B457-9A9644C838D5

Figs 8 , 26 , 31

Saropogon birdi Curran, 1931: 2.

#### References.

Curran 1931: 2 (key and original description); Martin and Wilcox 1965: 383 (catalog); Wilcox 1966: 129 (key to females); Fisher and Wilcox 1997: 4 (catalog).

**Figure 8.**
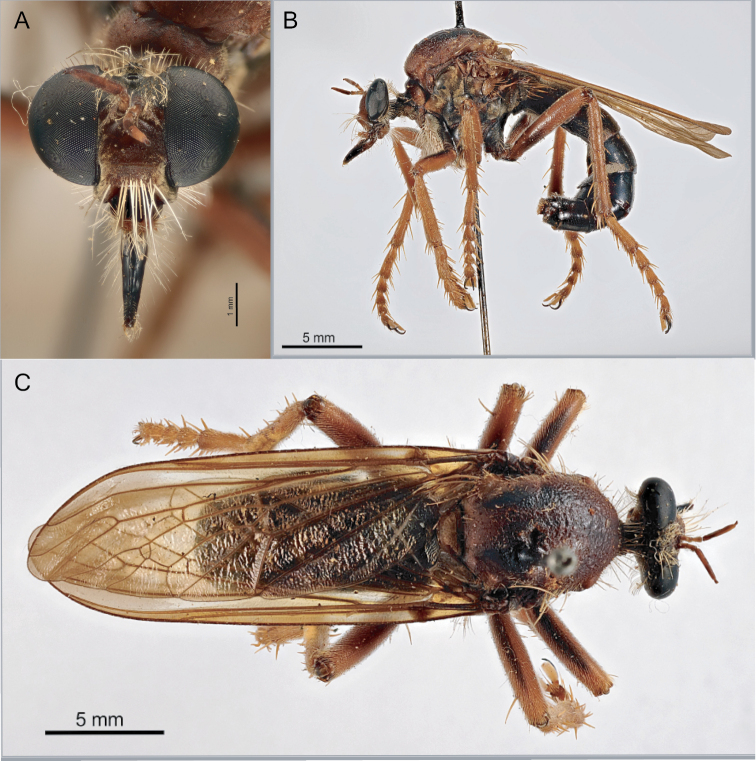
*Saropogonbirdi* Curran, 1931 Female holotype **A** anterior view **B** lateral view **C** dorsal view. Photograph provided by American Museum of Natural History.

#### Diagnosis.

Antennae mostly reddish except the style; base of palpi are black; femora black dorsally; coxal macrosetae yellowish; wings amber-colored with a tinge of brown apically; two apical scutellar macrosetae; abdomen mostly black. Body length 27 mm; wing length 15–21 mm. Flight time June.

Commonly confused with *Saropogonpritchardi* but *S.birdi* has black on the femora dorsum. Distinguished from *S.dispar* by having two apical scutellar macrosetae, and black basal segments of the palpi. *S.dispar* has four apical scutellar macrosetae and the female has orange basal segments of the palpi.

#### Distribution.

USA: Oklahoma.

#### Type material examined.

United States of America • 1 ♀, holotype; Oklahoma, Johnson County; 34°17'N, 96°37'W; 241 m; 20 June 1929; R. D. Bird; AMNH.

#### Comments.

We were only able to examine the holotype from images sent from the American Museum of Natural History where it is housed. We have been unable to find any other specimens of this species to examine.

### ﻿ Saropogon bryanti

Taxon classificationAnimaliaDipteraAsilidae

Wilcox, 1966

0E6E2D7C-DDD1-5B7E-AFDB-052EE974795B

**Figure 9.**
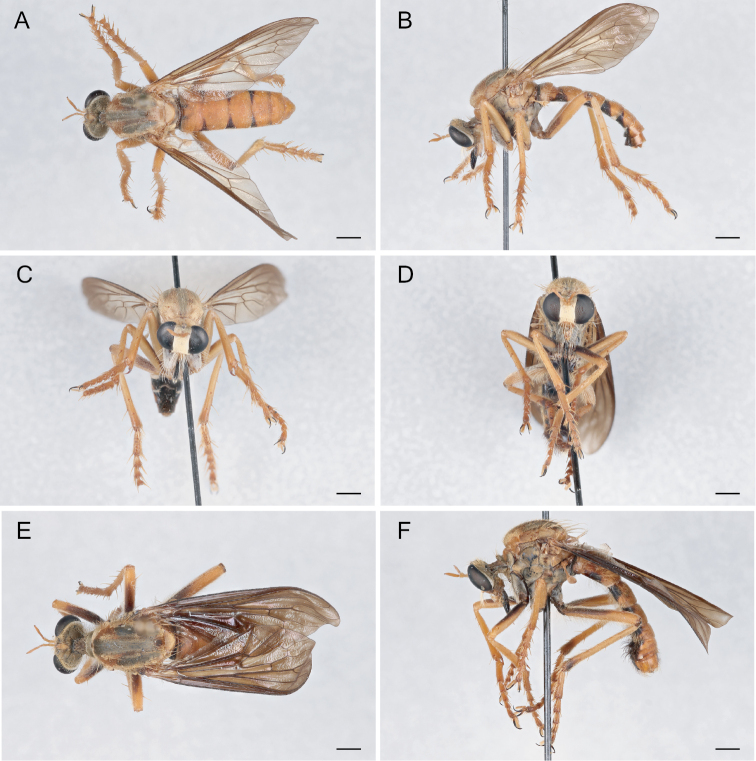
*Saropogonbryanti* Wilcox, 1966 Female (USNMENT01830074): **A** dorsal view **B** lateral view **C** anterior view; Male (USNMENT01830073): **D** anterior view **E** dorsal view **F** lateral view. Scale bars: 2 mm.

Figs 5C, D , 9 , 26 , 33

Saropogon bryanti Wilcox, 1966: 132.

#### References.

Wilcox 1966: 132 (key and original description); Fisher and Wilcox 1997: 4 (catalog).

#### Diagnosis.

Femur in both sexes reddish with black dorsal stripe; male and female face and frons with golden pubescence; scutum yellowish with the broad central stripe and elongated sub-lateral spots with gray pubescence. Male wing covered in microtrichia, female wing with microtrichia especially around veins Body length 16–19 mm; wing length 16–18 mm. Flight time June – August.

Distinguishable from *Saropogonhypomelas* by the face and frons being with golden pubescence and the extent of the black on the femora.

#### Distribution.

USA: Arizona, New Mexico, Texas; Mexico: Sonora.

#### Type material examined.

United States of America • 1 ♂, holotype; Arizona, Pima County, Baboquivari Canyon W. side Baboquivari Mts; 31°47'N, 111°37'W; 1124 m; 25–27 July 1952; H. B. Leech, J. W. Green; CASENT; Type no. 9278. • 1 ♀, allotype; same data as for holotype; CASENT; CASENT8427216 • 1 ♀, paratype; Arizona, Pima County, 8 mi. N. Tucson; 32°19'N, 110°58'W; 756 m; 11 June 1964; J. M. Davidson; USNM; USNMENT01830074.

#### Arizona material examined.

United States of America • 1 ♂; Cochise County, 7 mi. N. Mescal; 32°04'N, 110°26'W; 1097 m; 24 July 1966; F. G. Werner family; UAIC • 1 ♂; Cochise County, Portal; 31°54'N, 109°8'W; 1433 m; 02 June 1964; J. M. Davidson; USNM; USNMENT01830117 • 1 ♀; Cochise County, San Pedro River, 2 mi. E. Benson; 31°57'N, 110°16'W; 1073 m; 30 June 1963; J. C. Bequaert, P. H. Johnson; UAIC • 1 ?; Maricopa County, 3.2 mi. SE. of St. Johns, E. of Sierra Estrellas; 33°17'N, 112°10'W; 320 m; 07 July 1973; M. Kolner, J. Alcock; ASUHIC; ASUHIC139498, ASUHIC139499, ASUHIC139400, ASUHIC139401, ASUHIC139402, ASUHIC139403 • 33 ?; same collection data as for preceding; 10 July 1973; O. Francke, M. Kolner; ASUHIC; ASUHIC139404, ASUHIC139405, ASUHIC139406, ASUHIC139407, ASUHIC139408, ASUHIC139409, ASUHIC139410, ASUHIC139411, ASUHIC139412, ASUHIC139413, ASUHIC139414, ASUHIC139415, ASUHIC139416, ASUHIC139417, ASUHIC139418, ASUHIC139419, ASUHIC139420, ASUHIC139421, ASUHIC139422, ASUHIC139423, ASUHIC139424, ASUHIC139425, ASUHIC139426, ASUHIC139427, ASUHIC139428, ASUHIC139429, ASUHIC139430, ASUHIC139431, ASUHIC139432, ASUHIC139433, ASUHIC139434, ASUHIC139435, ASUHIC139436 •1 ♀; Maricopa County, 6 mi. N. of Scottsdale; 33°32'N, 111°55'W; 397 m; 07 September 1969; S. McCleve; UAIC • 3 ?; same collection data as for preceding; 22 July 1973; M. Kolner; ASUHIC; ASUHIC139437, ASUHIC139438, ASUHIC139439 • 2 ♂, 2 ♀; Maricopa County, 3.2 mi. SE. St. Johns, E. of Sierra Estrellas; 33°16'N, 112°13'W; 320 m; 10 July 1973; O. Francke, M. Kolner; CASENT; CASENT8427206, CASENT8427213, CASENT8427214, CASENT8427215 • 1 ?; Maricopa County, Granite Reef Dam; 33°30'N, 111°41'W; 401 m; 29 August 1964; J. M. Davidson; USNM; USNMENT01830106 • 1 ♂; Maricopa County; Sierra Mts.; 33°34'N, 111°42'W; 914–1219 m; 19 August 1924; A. A. Nichol; USNM; USNMENT01199077 • 2 ♂; Pima County, 4mi. E. Sahuarita; 31°57'N, 110°53'W; 861 m; 10 July, 1968; F. Werner, J. Burger, J. LaFage; UAIC • 1♀; Pima County 4 mi. SE. Sahuarita; 31°54'N, 110°54'W; 882 m; 17 July 1968; F. Werner, M. Noller; UAIC • 1 ♂; Pima County, 12 mi. N. Sasabe; 31°40'N, 111°58'W; 1134 m; 27 July 1973; E. M. Fisher; USNM; USNMENT01830118 • 1 ♀; Pima County, Santa Rita Experimenal Range Reserve; 31°49'N, 110°51'W; 1130 m; 21 July 1970; UAIC • 1 ♂; Pima County; 18 mi. W. Robles Jct.; 32°4'N, 111°37'W; 861 m; 30 August 1970; P. H. Sullivan; USNM; USNMENT01830108 • 2 ♂, 1 ♀; Pima County, 12 mi. n. Sasabe; 31°39'N, 111°32'W; 1122 m; 27 July 1973; E. M. Fisher; USNM; USNMENT01830105, USMENT01830073; CASENT; CASENT8427411 • 1 ?; Pima County, Madera Canyon; 31°44'N, 110°53'W; 1354 m; 23 July 1966; J. M. Davidson, M. A. Cazier; ASUHIC; ASUHIC0139493 • 1 ♂; Pima County, Range Res. 7 mi. N. Sahuarite; 32°05'N, 110°58'W; 785 m; 19 July 1979; F. Werner, Olson, Nygard; UAIC • 1 ♂, 1 ♀; Pima County, Saguaro National Monument Cast.; 32°17'N, 111°09'W; 829 m; 23 July 1978; B. lipa; UAIC • 1 ♂, 1 ♀; Pima County, Santa Catalina Mountains; 32°26'N, 110°47'W; 2776 m; 13 August 1940; E. C. Van Dyke; CASENT; CASENT8427209, CASENT8427210 • 1 ?; Pima County; Santa Rita Range Reserve; 31°43'N, 110°52'W; 1797 m; 15 July 1970; M. Cazier, J. Bigelow, L. Welch; ASUHIC; ASUHIC0139494 • 1 ?; same collection data as for preceding; M. Kolner, S. Szerlip; ASUHIC; ASUCIC0139495 • 2 ♂, 3 ♀; same collection data as for preceding; 31°49'N, 110°51'W; 1130 m; 06 July 1979; F. Werner, Olson, Nygard; UAIC; • 1 ♂; Pima County, Tucson; 32°13'N, 110°58'W; 724 m; 14 July 1947; USNM; USNMENT01199052 • 1 ♀; same collection data as for preceding; 18 July 1962; Wargo; UAIC • 1 ?; Pinal County, 12 mi. N. of Redington; 32°36'N, 110°29'W; 950 m; 20 July 1966; J. M. Davidson, M. A. Cazier; ASUHIC; ASUHIC0139492 • 1 ♂; Pinal County, Apache Junction; 33°25'N, 111°34'W; 512 m; 30 July 1929; UAIC • 5 ♂, 2 ♀; Santa Cruz County, Santa Rita Mtns., Madera Canyon; 31°47'N, 110°55'W; 1049 m; 14–22 July 1971; D. G. Marqua, P. Sullivan; USNM; USNMENT0183007, USNMENT01830110, USNMENT01830111, USNMENT01830112, USNMENT01830113, USNMENT01830114, USNMENT01830115 • 1 ♂; same collection data as for preceding; 1503 m; 01 August 1960; S. L. Wood, J. B. Karren, H. Shurtleff; BYU; BYUC215968 • 3 ♂, 5 ♀; same collection data as for preceding; 12 July 1973; D. G. Marqua; CASENT; CASENT8427208; USNM; USNMENT01830116, USNMENT01830121, USNMENT01830122, USNMENT01830123, USNMENT01830124, USNMENT01830125, USNMENT01830126 • 1 ♀; Yavapai County, Congress; 34°9'N, 112°51'W; 931 m; 20 July 1930; T. F. Winburn, R. H. Painter; CASENT; CASENT8427207.

#### Other material examined.

Suppl. material 1.

#### Comments.

One specimen we examined was from Iowa (CASENT8427218, Suppl. material 1), though the species seems to be identified correctly, this is still an unusual occurrence and may be a mistake, so it is not included in the known distribution for this species. Photographs of the *Saropogonbryanti* holotype can be found at: https://monarch.calacademy.org/taxa/index.php?tid=679454.

### ﻿ Saropogon combustus

Taxon classificationAnimaliaDipteraAsilidae

Loew, 1874

01361475-61B9-55EB-A0DD-1EA8046F43C1

**Figure 10.**
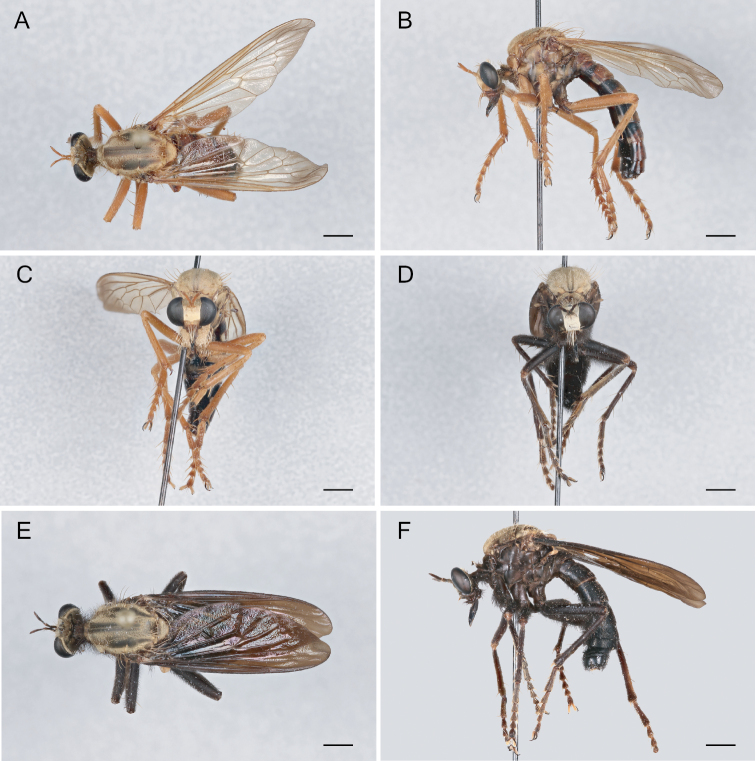
*Saropogoncombustus* Loew, 1874 Female (USNMENT01819131): **A** dorsal view **B** lateral view **C** anterior view; Male (USNMENT01819138): **D** anterior view **E** dorsal view **F** lateral view. Scale bars: 2 mm.

Figs 4C, D , 5E, F , 10 , 26 , 34

Saropogon combustus Loew, 1874: 373.
Saropogon adustus Loew, 1874: 375, junior synonym.

#### References.

Osten-Sacken 1874:185 (catalog); Back 1909: 347 (key and redescription); Curran 1930: 2 (key), 1931: 2 (key and notes); Martin and Wilcox 1965: 383 (catalog); Wilcox 1966: 129 (key); Fisher and Wilcox 1997: 4 (catalog).

#### Diagnosis.

This species is sexually dimorphic: males mostly black, wings brown, four scutellar bristles; females reddish, wings yellowish, anterior corners of T2–5 black. Body length 13–19 mm; wing length 14–17 mm. Flight time May – October.

The male is easily distinguished from *Saropogonfletcheri* and *S.pritchardi* because it is significantly darker and more robust than the other males. The female is a bit more challenging but can be separated from *S.fletcheri* because it does not have the black anterior bands on its abdomen. The female *S.pritchardi* also has significantly darker wings than *S.combustus* which is pale brown and darker apically.

#### Distribution.

USA: Colorado, Kansas, Nebraska, New Mexico, Oklahoma, Texas, SimpleMappr: https://www.simplemappr.net/map/16981.

#### Type material examined.

United States of America • 1 ♂, holotype; Loew; photographed pinned specimen; MCZ; Type 12819 • 1 ♀; Loew; MCZ; Type 12818.

#### Other material examined.

Suppl. material 1.

#### Comments.

The holotypes of both *Saropogoncombustus* and *S.adustus* (junior synonym) are in the Museum of Comparative Zoology at Harvard University. The collection provides photos of the types on their website MCZBase: https://mczbase.mcz.harvard.edu/MediaSearch.cfm?action=search&media_id=99135,99136,99137,99138,99139 and https://mczbase.mcz.harvard.edu/MediaSearch.cfm?action=search&media_id=99130,99131,99132,99133,99134.

### ﻿ Saropogon coquillettii

Taxon classificationAnimaliaDipteraAsilidae

Back, 1909

21E25A9A-57D5-5E45-ABE5-37D16FA02E37

Figs 11 , 26 , 32

Saropogon coquillettii Back, 1909: 348.
Saropogon coquilletti auctt: common misspelling.

#### References.

Back 1909: 348 (original description and key); Curran 1930: 2 (key), 1931: 2 (key); Martin and Wilcox 1965: 383 (catalog); Fisher and Wilcox 1997: 4 (catalog).

**Figure 11.**
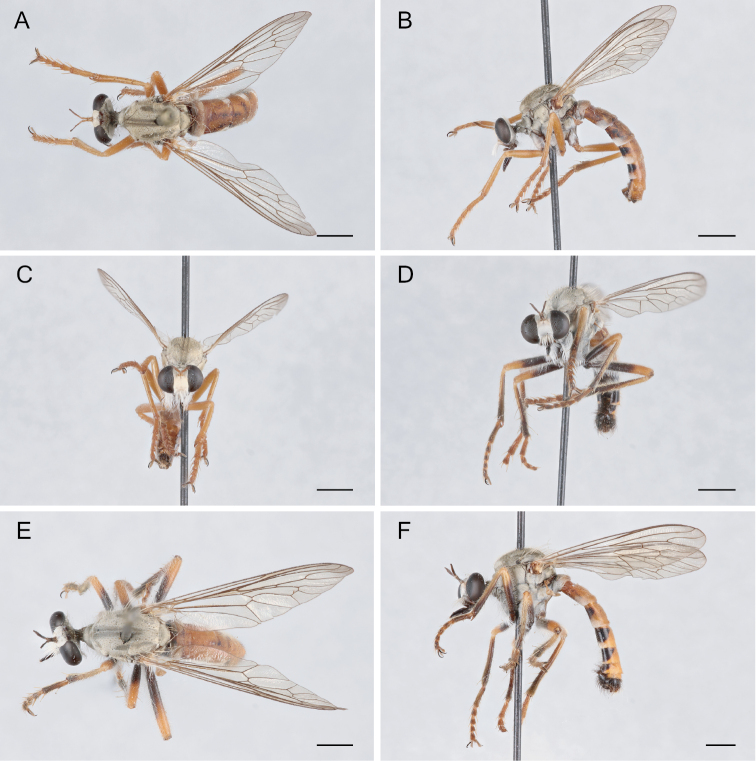
*Saropogoncoquillettii*Back 1909 Female (USNMENT01830076): **A** dorsal view **B** lateral view **C** anterior view; Male (USNMENT01830075): **D** anterior view **E** dorsal view **F** lateral view. Scale bars: 2 mm.

#### Diagnosis.

*Saropogoncoquillettii* is similar to *S.semiustus*, *S.hyalinus*, and *S.luteus*, but can be separated from them because it has four scutellar bristles instead of two. It has nearly hyaline wings with only a tinge of color apically and is more slender than *Saropogoncombustus* and *S.dispar*. Body and wing length 14–16 mm. Flight time May – October.

#### Distribution.

USA: Arizona, New Mexico, Texas; Mexico: Sonora, SimpleMappr: https://www.simplemappr.net/map/16982.

#### Type material examined.

United States of America • 1♂, holotype; New Mexico, Doña Ana County, Las Cruces; 32°28'N, 106°52'W; 1247 m; Aug 1923; Townsend; USNM; USNMENT01199124 • 1♂, 1♀, topotype; same locality data as holotype; 28 Jul; Townsend; USNM; USNMENT01199038, USNMENT01199017.

#### Arizona material examined.

United States of America • 2♂, 4♀; Comal County, Cañon Lake; 33°32'N, 111°27'W; 631 m; 02 September 1935; F. H. Parker; USNM; USNMENT01199096, USNMENT01199088, USNMENT01199036, USNMENT01199092, USNMENT01199119, USNMENT01199045 • 1♀; Gila County, Globe; 32°22'N, 110°51'W; 1237 m; August; D. K. Duncan; USNM; USNMENT01518366 • 1♀; same collection data as for proceeding; 24 August 1957; F. H. Parker; UAIC • 2♂, 1♀, 1?; Gila County, San Carlos Lake; 33°11'N, 110°28'W; 749 m; August; D. K. Duncan; CASENT; CASENT8427290, CASENT8427291; USNM; USNMENT01199029, USNMENT01199043 • 1♂; Maricopa County, Higley; 33°18'N, 111°42'W; 398 m; 24 July 1917; E. G. Holt; USNM; USNMENT01819460 • 1♂; Maricopa County, Phoenix; 33°26'N, 112°04'W; 334 m; 01 August 1960; R. E. Rice; USNM; USNMENT01830392 • 1♀; Pima County, 30 mi. SE Ajo; 32°07'N, 112°26'W; 612 m; 30 July 1966; R. L. Brumley; BME; BMEP0280586 • 10♂; Pima County, Picacho Pass; 32°39'N, 111°23'W; 555 m; 13 September 1954; J. C. Hall; BME; BMEP0280451, BMEP0280590, BMEP0280593, BMEP0280599, BMEP0280616, BMEP0280594, BMEP0280619, BMEP0280534, BMEP0280533, BMEP0280618 • 1♂, 2♀, 1?; Pinal County, 15 mi. S. of Florence; 32°50'N, 111°21'W; 631 m; 20 August 1949; F. H. Parker; USNM; USNMENT01199016, USNMENT01199056, USNMENT01199073 • 1♀; Pinal County; 32°48'N, 111°17'W; 619 m; 18 August 1940; E. R. Leach; CASENT; CASENT8427292 • 3♀; Pinal County, Mt. Superstition near Higley; 33°28'N, 111°11'W; 1424 m; 24 July 1917; E. G. Holt; USNM; USNMENT01819540, USNMENT01819520, USNMENT01819530.

#### Other material examined.

Suppl. material 1.

#### Comments.

This species is often misspelled (e.g., Curran 1930, 1931) as *Saropogoncoquilletti*, but the original description states *S.coquillettii*. Photographs of the holotype can be viewed at: http://n2t.net/ark:/65665/326f621b6-964b-4453-8fb5-715b5480ab6f.

### ﻿ Saropogon dispar

Taxon classificationAnimaliaDipteraAsilidae

Coquillett, 1902

30374D0F-F14C-5158-A5C6-630F29CD8C3C

**Figure 12.**
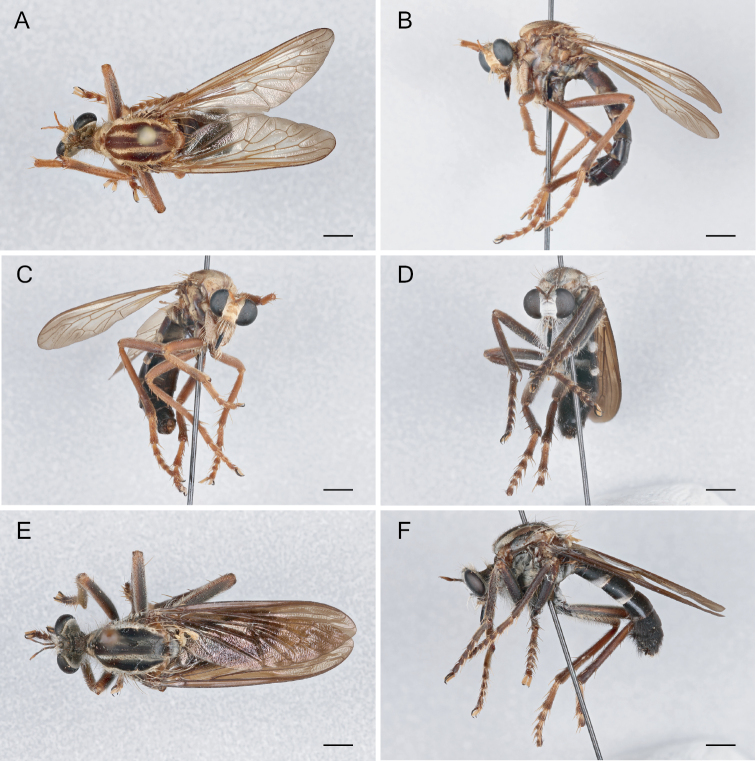
*Saropogondispar* Coquillett, 1902 Female (UCBMEP0280509): **A** dorsal view **B** lateral view **C** anterior view; Male (UCBMEP0280508): **D** anterior view **E** dorsal view **F** lateral view. Scale bars: 2 mm.

Figs 5G, H , 12 , 32

Saropogon dispar Coquillett, 1902: 139.

#### References.

Back 1909: 349 (key and redescription); Curran 1930: 2 (key), 1931: 2 (key and notes); Martin and Wilcox 1965: 383 (catalog); Wilcox 1966: 129 (key); Fisher and Wilcox 1997: 4 (catalog).

#### Diagnosis.

This species is sexually dimorphic: males with brown wings, black mesonotum and legs, brownish tibiae and tarsi; females with yellowish wings, brown mesonotum, reddish legs, distally blackish prothoracic and mesothoracic femora. Body length 20–23 mm; wing length 18–21 mm. Flight time May – August.

*Saropogondispar* may be confused with *S.hypomelas* or *S.bryanti* but it is a significantly darker species than either.

#### Distribution.

USA: Oklahoma, Texas, SimpleMappr: https://www.simplemappr.net/map/16983.

#### Type material examined.

United States of America • 1♂, holotype; Texas, DeWitt County, Cuero; 29°05'N, 97°17'W; 57 m; 06 Jun.; USNM; USNMENT01199066

#### Other material examined.

Suppl. material 1.

#### Comments.

Bromley (1934) states “*Saropogondispar* is by far the most noxious species in bee-yards in the San Antonio region.” See Table 1 for prey records. Access photographs of the holotype at http://n2t.net/ark:/65665/33098b0bf-d97f-4b92-9141-eaa52cd9f59a.

### ﻿ Saropogon fletcheri

Taxon classificationAnimaliaDipteraAsilidae

Bromley, 1934

7FA8918C-3272-5AE7-BF81-45F59FF2290E

Figs 13 , 26 , 34

Saropogon fletcheri Bromley, 1934: 91.

#### References.

Bromley 1934: 91 (original description); Martin and Wilcox 1965: 383 (catalog); Wilcox 1966: 130 (key); Fisher and Wilcox 1997: 4 (catalog).

**Figure 13.**
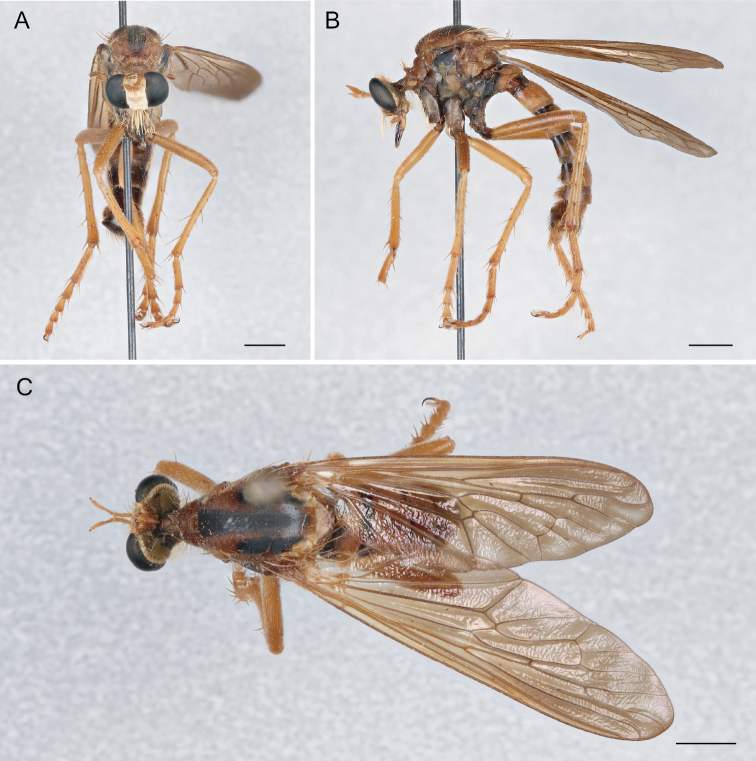
*Saropogonfletcheri* Bromley, 1934 Male (UCBMEP0280504): **A** anterior view **B** lateral view **C** dorsal view. Scale bars: 2 mm.

#### Diagnosis.

This species is sometimes similar to *Saropogondispar* but both sexes are reddish and the femora lack black. Scutellum has four reddish bristles; and wings are pale reddish brown. Body length 24–17 mm; wing length 11–14 mm. Flight time April – October.

#### Distribution.

USA: Arizona, Texas, SimpleMappr: https://www.simplemappr.net/map/16984.

#### Type material examined.

United States of America • 1♂, holotype; Texas, Comfort; 29°58'N, 98°54'W; 19 July 1921; R. K. Fletcher; TAMUIC.

#### Arizona material examined.

United States of America • 1♀; Maricopa County, Morales; 34°02'N, 111°05'W; 1496 m; 27 August 1913; W. D. Pierce; USNM; USNMENT01819450.

#### Other material examined.

Suppl. material 1.

### ﻿ Saropogon hyalinus

Taxon classificationAnimaliaDipteraAsilidae

Coquillett, 1904

4D6BFA2E-0BD0-55C5-AF0B-B0131821AE33

Figs 14 , 26 , 32

Saropogon hyalinus Coquillett, 1904: 185.

#### References.

Back 1909: 351 (key and short redescription); Curran 1930: 2 (key), 1931: 2 (key); Martin and Wilcox 1965: 383 (catalog); Wilcox 1966: 129 (key); Fisher and Wilcox 1997: 4 (catalog).

**Figure 14.**
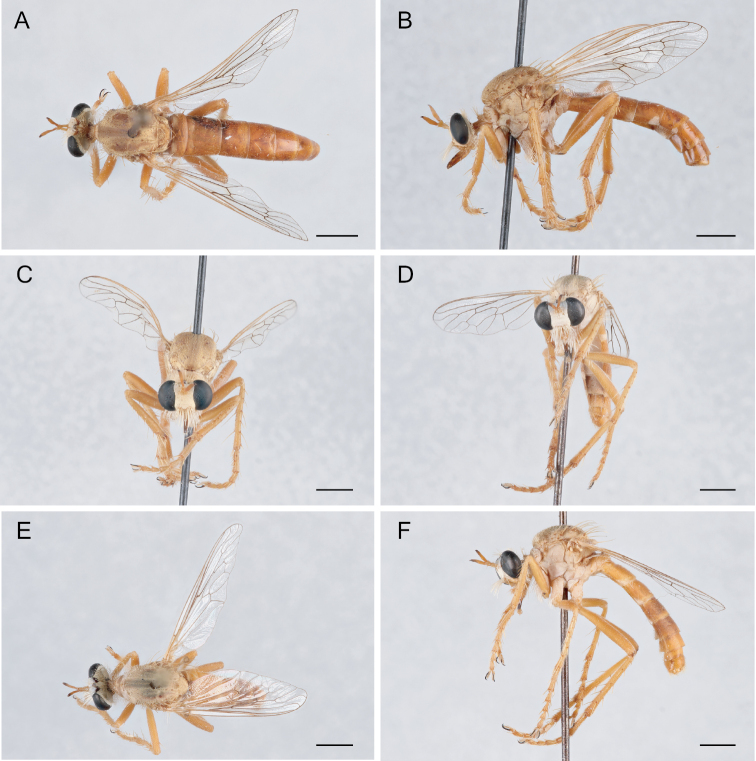
*Saropogonhyalinus* Coquillett, 1904 Female (USNMENT01830078): **A** dorsal view **B** lateral view **C** anterior view; Male (UCBMEP0280500): **D** anterior view **E** dorsal view **F** lateral view. Scale bars: 2 mm.

#### Diagnosis.

This species is similar to *Saropogonluteus* except the wings are pure hyaline, and the scutum is densely with yellowish pubescence, with gray pubescent median stripe and elongated sub-lateral spots, crossing the transverse suture. Body length 13–17 mm; wing length 9–11 mm. Flight time May – September.

#### Distribution.

USA: California, SimpleMappr: https://www.simplemappr.net/map/16985.

#### Type material examined.

United States of America • 1 ♀, holotype; California, Los Angeles County; 34°03'N, 118°14'W; 97 m; Coquillett; USNM; USNMENT01199005.

#### Other material examined.

Suppl. material 1.

#### Comments.

You can access photographs of the holotype here: http://n2t.net/ark:/65665/308595f92-7180-42d6-a5ed-8be56e3423d4.

### ﻿ Saropogon hypomelas

Taxon classificationAnimaliaDipteraAsilidae

(Loew, 1866)

452D2C44-6DDA-54D3-B9AC-971EC6225EA4

**Figure 15.**
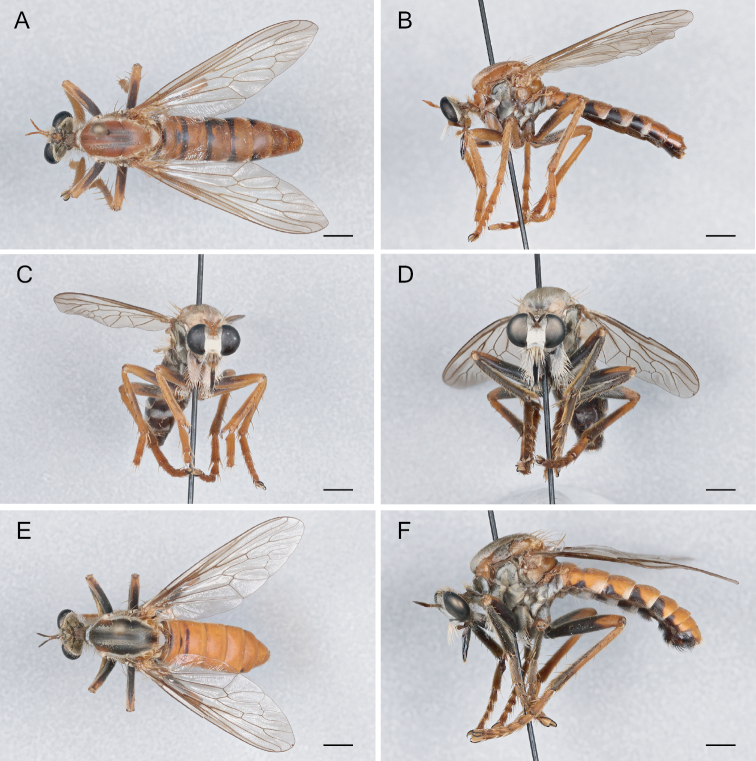
*Saropogonhypomelas* Loew, 1866 Female (USNMENT01830080): **A** dorsal view **B** lateral view **C** anterior view; Male (UCBMEP0280599): **D** anterior view **E** dorsal view **F** lateral view. Scale bars: 2 mm.

Figs 5I, J , 15 , 26 , 33

Diogmites hypomelas Loew, 1866: 24 [= Saropogonhypomelas (Loew)].

#### References.

Loew 1866: 24 (as *Diogmites*); Martin and Wilcox 1965: 383 (catalog); Wilcox 1966: 133 (key and translation of original description); Fisher and Wilcox 1997: 4 (catalog).

#### Diagnosis.

A large, sexually dimorphic species. Male with legs reddish, femur, sometimes tibia, black; face and frons with white pubescence; female femur proximally black or with proximal black dorsal stripe; face and frons with golden pubescence; both sexes with scutum with yellowish-gray pubescence, median stripe with brown pubescence. Body length 17–27 mm; wing length 17–18 mm. Flight time April – September.

#### Distribution.

USA: Arizona, New Mexico, Texas; Mexico: Coahuila, Nuevo Leon, SimpleMappr: https://www.simplemappr.net/map/16986.

#### Type material examined.

**United Staes of America** • 1 ♀, syntype, New Mexico; 34°17'N, 106°17'W; Loew; MCZ; MCZ-ENT00012822.

#### Arizona material examined.

United States of America • 1 ♀; Maricopa County, 3 mi. N. Gila Bend; 32°58'N, 112°42'W; 205 m; 27 July 1969; H. A. Smith; CASENT; CASENT8427317 • 1 ♀; Pima County, Madera Canyon; 31°43'N, 110°52'W; 1503 m; 14 July 1980; T. L. McKenzie; USNM; USNMENT01830394 • 1 ?; Pima County, Santa Rita Mtns. Madera Canyon; 31°43'N, 110°52'W; 1503 m; 13 September 1964; R. H. Crandall; LACM; LACMENT579085

#### Other material examined.

Suppl. material 1.

#### Comments.

Martin and Wilcox (1965) included the name *Saropogonhypomelas* in their catalog. They did not state it as a new change, and the author who first transferred *Diogmiteshypomelas* to *Saropogon*, is still unknown. Wilcox (1966) mentions receiving correspondence from Bromley in 1936 saying that after examining the type, he believed that it belonged in *Saropogon* Loew.

The syntype can be viewed at MCZBase: https://mczbase.mcz.harvard.edu/guid/MCZ:Ent:12822. The syntypes were listed under the name *Deromyiahypomelas* but have since been changed to the current valid name.

iNaturalist lists a record of *Saropogonhypomelas* from Oklahoma (https://www.inaturalist.org/observations/90489061) This photographed specimen evidently is correctly identified and would extend the known range for this species.

### ﻿ Saropogon laparoides

Taxon classificationAnimaliaDipteraAsilidae

Bromley, 1951

EC3855C8-8F3D-5B8F-A84E-DCCBE01F97BF

Figs 16 , 26 , 32

Saropogon laparoides Bromley, 1951: 14.

#### References.

Martin and Wilcox 1965: 383 (catalog); Wilcox 1966 (junior synonym *S.sculleni* is described and keyed); Fisher and Wilcox 1997: 4 (catalog).

**Figure 16.**
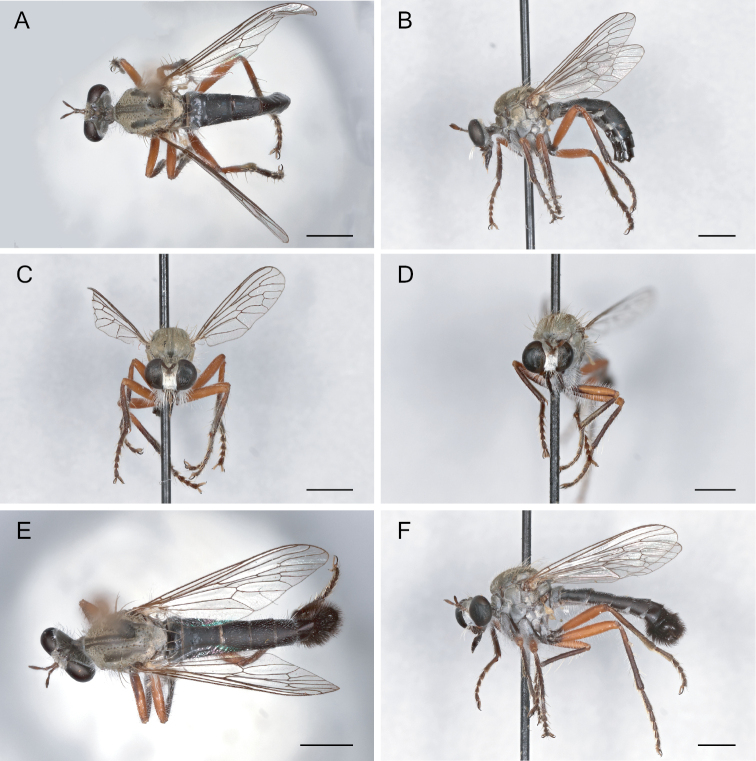
*Saropogonlaparoides* Bromley, 1951 Female (USNMENT01819592): **A** dorsal view **B** lateral view **C** anterior view; Male (USNMENT01819567): **D** anterior view **E** dorsal view **F** lateral view. Scale bars: 2 mm.

#### Diagnosis.

A small, dark species with hyaline wings and white coxal bristles. Females with mostly reddish legs with the tips of the tibiae and tarsi blackish and scutum with gray pubescence; Male femora mostly reddish, prothoracic and mesothoracic femora black dorsally, tibiae and tarsi blackish and mesonotum with yellowish gray pubescence. Male terminalia with many black setae. Body length 12–16 mm; wing length 8–9 mm. Flight time July – August.

#### Distribution.

USA: Texas, SimpleMappr: https://www.simplemappr.net/map/16987.

#### Type material examined.

United States of America • 1 ♀, holotype; Texas, Presidio County, Presidio; 29°33'N, 104°22'W; 787 m; 04 Aug. 1929; AMNH • 1 ♀, paratype; Texas, Presidio County, Chinati Mtns; 29°54'N, 104°27'W; 1924 m; 04 Aug. 1924; E. R. Tinkham; USNM; USNMENT01819182

#### Other material examined.

Suppl. material 1.

#### Comments.

According to Bromley (1951), this species resembles an African Dasypogoninae genus, *Meolapharus* [sic] (= *Neolaparus*, junior synonym of the widespread genus *Pegesimallus* (Londt, 1980)).

### ﻿ Saropogon luteus

Taxon classificationAnimaliaDipteraAsilidae

Coquillett, 1904

F6371477-52F4-58CF-A680-A82C20E190CF

**Figure 17.**
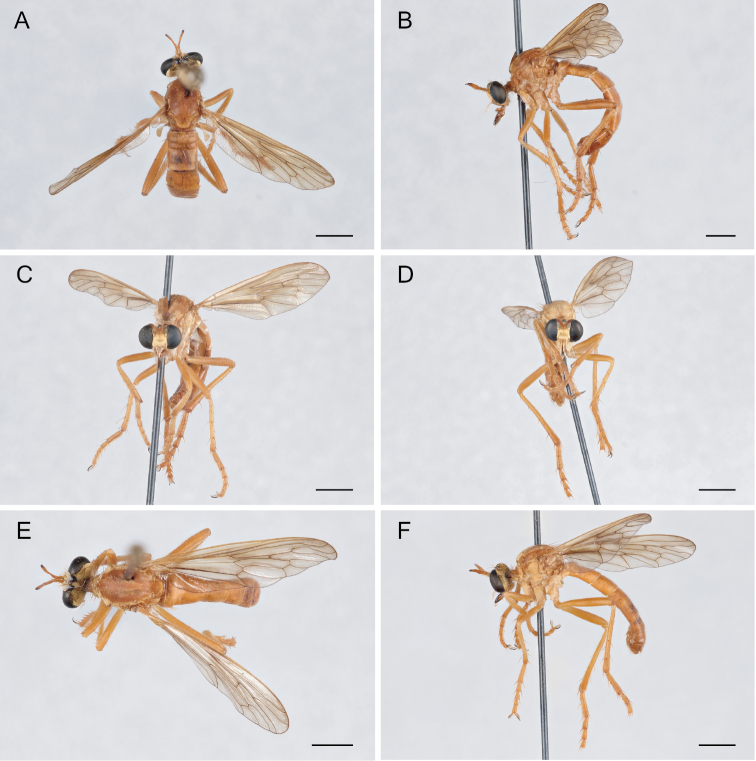
*Saropogonluteus* Coquillett, 1904 Female (UCBMEP0073792): **A** dorsal view **B** lateral view **C** anterior view; Male (UCBMEP0073760): **D** anterior view **E** dorsal view **F** lateral view. Scale bars: 2 mm.

Figs 5K, L , 17 , 26 , 33

Saropogon luteus Coquillett, 1904: 185.
Saropogon rufus Back 1904: 290, junior synonym.

#### References.

Back 1909: 351 (key and redescription); Curran 1930: 2 (key); Curran 1931: 2 (key); Martin and Wilcox 1965: 383 (catalog); Wilcox 1966: 130 (key); Fisher and Wilcox 1997: 4 (catalog).

#### Diagnosis.

This species is the most likely one to be confused with *Saropogonpyrodes* sp. nov. because of its reddish color. They are easily distinguished by the entire anepisternum of *Saropogonluteus* being with gold pubescence instead of white as in *S.pyrodes* sp. nov. *Saropogonluteus* also has small, with gray pubescent spots on the posterior corners of the tergites. This species is almost exclusively found in California. Body length 11–17 mm; wing length 8–10 mm. Flight time May – September.

#### Distribution.

USA: California; Mexico: Baja California SimpleMappr: https://www.simplemappr.net/map/16988.

#### Type material examined.

United States of America • 1♀, holotype; California, Los Angeles County; 34°03'N, 118°14'W; 97 m; Coquillett; USNM; USNMENT01199100.

#### Other material examined.

Suppl. material 1.

#### Comments.

Photographs of the holotype are available here: http://n2t.net/ark:/65665/338f15b33-0872-416f-8a58-277c87bb8142. The holotype of *Saropogonrufus* (junior synonym to *S.luteus*) is in the Museum of Comparative Zoology at Harvard University. Photographs of this specimen are available here: https://mczbase.mcz.harvard.edu/guid/MCZ:Ent:7583.

### ﻿ Saropogon mohawki

Taxon classificationAnimaliaDipteraAsilidae

Wilcox, 1966

ADF8D32A-9368-50B1-868A-02F3A5F5AE3C

Figs 18 , 26 , 34

Saropogon mohawki Wilcox, 1966: 134.

#### References.

Wilcox 1966: 134 (key and original description); Fisher and Wilcox 1997: 4 (catalog).

#### Diagnosis.

Wings completely hyaline, the posterior corners of T2–4 with gray pubescence, the anterior corners of T4 and 5 (sometimes T4–6) with black spots; legs pale-colored in both sexes but sometimes femora blackish basally in male. This species is mostly easily confused with *Saropogoncoquillettii*; the main differences are the extent of abdominal markings and the lack of wing microtrichia. Body length 10–13 mm; wing length 11–15 mm. Flight time May – October.

**Figure 18.**
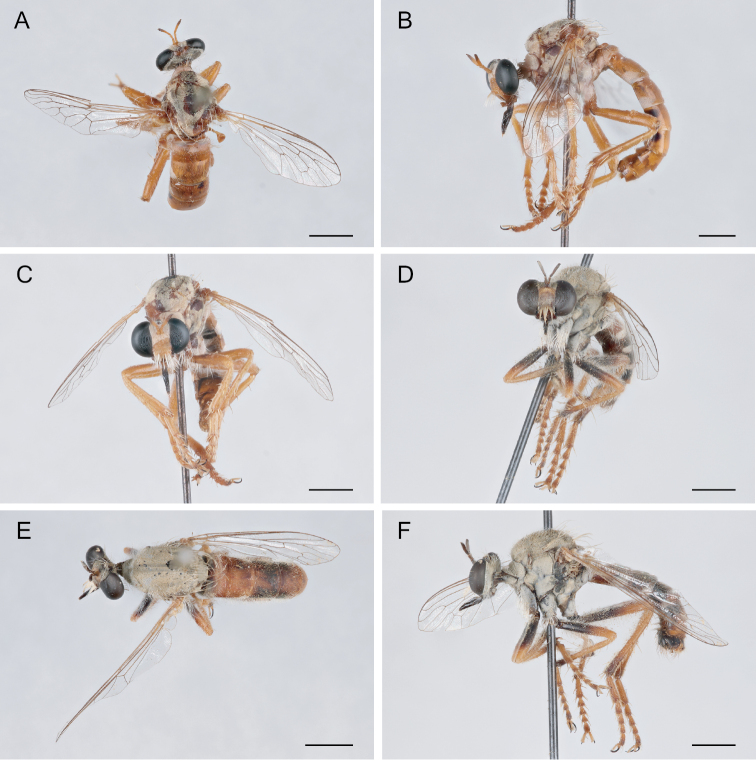
*Saropogonmohawki* Wilcox, 1966 Female paratype (UCBMEP0003173): **A** dorsal view **B** lateral view **C** anterior view; Male (UCBMEP0003175): **D** anterior view **E** dorsal view **F** lateral view. Scale bars: 2 mm.

#### Distribution.

USA: Arizona, California, Nevada, Utah; Mexico: Baja California, Sonora, SimpleMappr: https://www.simplemappr.net/map/16989.

#### Type material examined.

United States of America • 1 ♂, holotype; Arizona, Yuma County, Mohawk; 32°43'N, 113°45'W; 166 m; 16 Jul 1962; J. Wilcox; CASENT; Type No. 9279 • 1 ♀, paratype; Arizona, Yuma County, 25 mi. SE. Parker; 33°51'N, 114°3'W; 361 m; 05 Sep 1964; J. M. Davidson; USNM; USNMENT01830250 • 1 ♂, paratype; California, San Bernardino, Baker; 35°16'N, 116°4'W; 286 m; 24 Jun 1930; F. H. Wymore; BMEC; UCBMEP0003174.

#### Arizona material examined.

United States of America • 1 ♀; La Paz County, Ehrenberg; 33°36'N, 114°31'W; 91 m; 27 Aug. 1938; F. H. Parker; UAIC • 1 ?; Maricopa County, 1.6 mi. SE. of Barnes Butte, near Papago Park; 33°27'N, 111°56'W; 378 m; 23 June 1973; M. Kolner; ASUHIC; ASUHIC0139654 • 1 ?; same collection data as for preceding; 20 July 1973; M. Kolner; ASUHIC; ASUHIC0139653 • 2 ?; same collection data as for preceding; 26 July 1973; M. Kolner; ASUHIC; ASUHIC0139655, ASUHIC0139656 • 1 ♀; Maricopa County, Cave Creek; 33°50'N, 111°57'W; 689 m; 08 June 1947; F. H. Parker, USNM; USNMENT01819560 • 3 ♂, 4 ♀; Maricopa County, Gila River 10 km S. Arlington; 33°13'N, 112°45'W; 200 m; 4–14 August 2010; M. E. Irwin; UAIC • 2 ♂; same collection data as for preceding; 14–21 August 2010; M. E. Irwin; UAIC • 4 ♂, 3 ♀; same collection data as for preceding; 15–31 July 2010; M. E. Irwin; UAIC • 1 ♂, 6 ♀; same collection data as for preceding; 1–7 June 2010; M. E. Irwin; UAIC • 1 ♀; same collection data as for preceding; 3–7 June 2010; M. E. Irwin; UAIC • 1 ?; Maricopa, S. Mtn. Park, 1.4 mi. W. of Elliot Rd. and Freeway; 33°20'N, 112°04'W; 539 m; 16 July 1972; M. Kolner; ASUHIC; ASUHIC0139657 • 1 ♂; Mariposa County, 6 mi. W. Gila Bend; 32°56'N, 112°49'W; 220 m; 09 September 1961; G. I. Stage; CASENT; CASENT8427321 • 2 ♀; Pima County, Organ Pipe Cac. N. M. Quitobaquito; 32°01'N, 112°49'W; 524 m; 07 April 1968; J. Gruwell; USNM; USNMENT01830276, USNMENT01830277 • 1 ♀; Pima County, Organ Pipe Cactus NM Quitobaquito Springs; 31°56'N, 113°01'W; 326 m; 27 August 1983; Kinglsey, Bailowatz; UAIC • 1 ♀; Yuma County, 1 mi. NW Aztec; 32°50'N, 113°27'W; 140 m; 31 August 1979; E. M. Fisher; USNM; USNMENT01830254 • 1 ♀; Yuma County, 13 mi. W. Hope; 33°42'N, 113°55'W; 380 m; 30 August 1979; E. M. Fisher; USNM; USNMENT01830253 • 1 ♂, 1 ♀; Yuma County, 25 mi. SE Parker; 33°51'N, 114°3'W; 361 m; 05 September 1964; J. M. Davidson; USNM; USNMENT01830250 • 1 ?; Yuma County, 37 mi. S. of Quartzsite; 33°07'N, 114°13'W; 409 m; 26 July 1966; J. M. Davidson, M. A. Cazier; ASUHIC; ASUHIC0139641 • 2 ?; Yuma County, 37 mi. S. of Quartzsite; 33°07'N, 114°13'W; 409 m; J. M. Davidson, M. A. Cazier; ASUHIC; ASUHIC0139647, ASUHIC0139648 • 1 ?; Yuma County, 6 mi. SE. of Parker; 34°05'N, 114°12'W; 208 m; 09 July 1966; J. M. Davidson, M. A. Cazier; ASUHIC0139642 • 1 ?; Yuma County; 8 mi. SE. of Parker; 34°04'N, 114°11'W; 262 m; 29 May 1966; S. A. Gorodenski; ASUHIC; ASUHIC0139640 • 1 ♀; Yuma County, Mohawk; 32°43'N, 113°45'W; 166 m; 26 August; J. Wilcox; CASENT; CASENT8427320.

#### Other material examined.

Suppl. material 1.

#### Comments.

Photographs of the holotype can be viewed at: https://monarch.calacademy.org/taxa/index.php?tid=679456.

### ﻿ Saropogon nitidus

Taxon classificationAnimaliaDipteraAsilidae

Wilcox, 1966

1B4CC9CE-2D1D-5530-B5F2-0D279AF9C982

Figs 19 , 31

Saropogon nitidus Wilcox, 1966: 135.

#### References.

Wilcox 1966: 135 (key and original description); Fisher and Wilcox 1997: 4 (catalog).

#### Diagnosis.

This species can be easily distinguished from others in the region by a shining black non-pubescent spot on the anterior half of the anepisternum and katepisternum. The male has yellowish red femora with black tibiae and tarsi; the posterior corners of T2–5 (males) and T2–4 (females) are with white pubescence; legs in female are yellowish. Body length 12–14 mm; wing length 8–10 mm. Flight time May – October.

**Figure 19.**
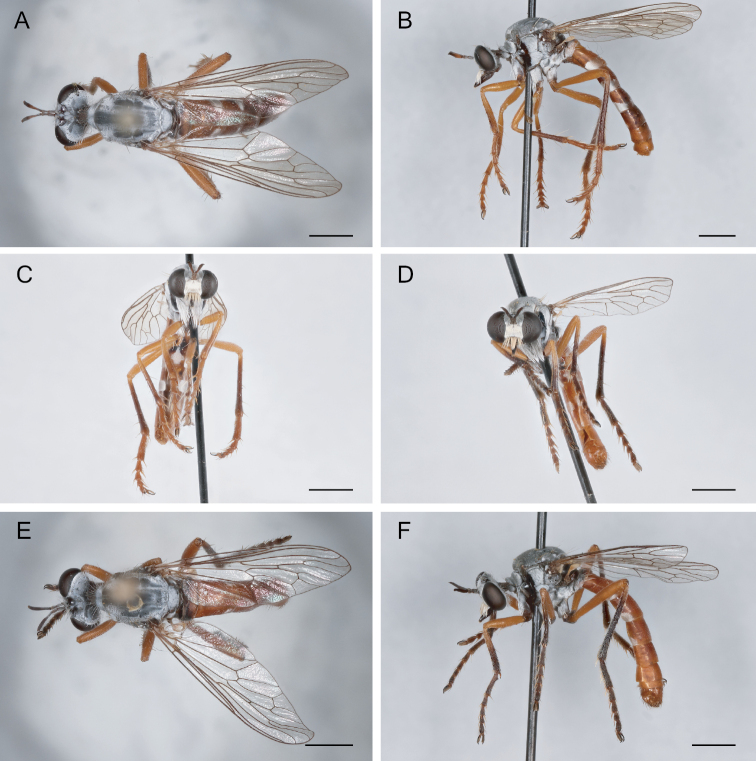
*Saropogonnitidus* Wilcox, 1966 Female (USNMENT01830081): **A** dorsal view **B** lateral view **C** anterior view; Male (UCBMEP0280497): **D** anterior view **E** dorsal view **F** lateral view. Scale bars: 2 mm.

#### Distribution.

USA: New Mexico, Texas; Mexico: Chihuahua, Coahuila, SimpleMappr: https://www.simplemappr.net/map/16990.

#### Type material examined.

United States of America • 1 ♂, holotype; Texas, Brewster County, Lajitas; 29°15'N, 103°46'W; 714 m; 04 Sep 1961; J. E. Gillaspy; CASENT; Type No. 9280.

#### Other material examined.

Suppl. material 1.

#### Comments.

Photographs of holotype can be found at: https://monarch.calacademy.org/taxa/index.php?tid=679457.

### ﻿ Saropogon pritchardi

Taxon classificationAnimaliaDipteraAsilidae

Bromley, 1934

C84D2C01-0D4B-5B69-AD0C-3778F5459C5E

Figs 20 , 33

Saropogon pritchardi Bromley, 1934: 90.

#### References.

Martin and Wilcox 1965: 383 (catalog); Wilcox 1966: 129 (key); Fisher and Wilcox 1997: 4 (catalog).

#### Diagnosis.

This is a large species but slightly smaller and more slender than *Saropogondispar*. The wings are proportionately longer and broader than those of *S.dispar* and the legs are uniformly reddish without any dark markings. Wings and abdomen are black, the thorax with yellowish pubescence, and scutellum has two pale-colored bristles. Body length 20–23 mm; wing length 16–18 mm. Flight time July.

**Figure 20.**
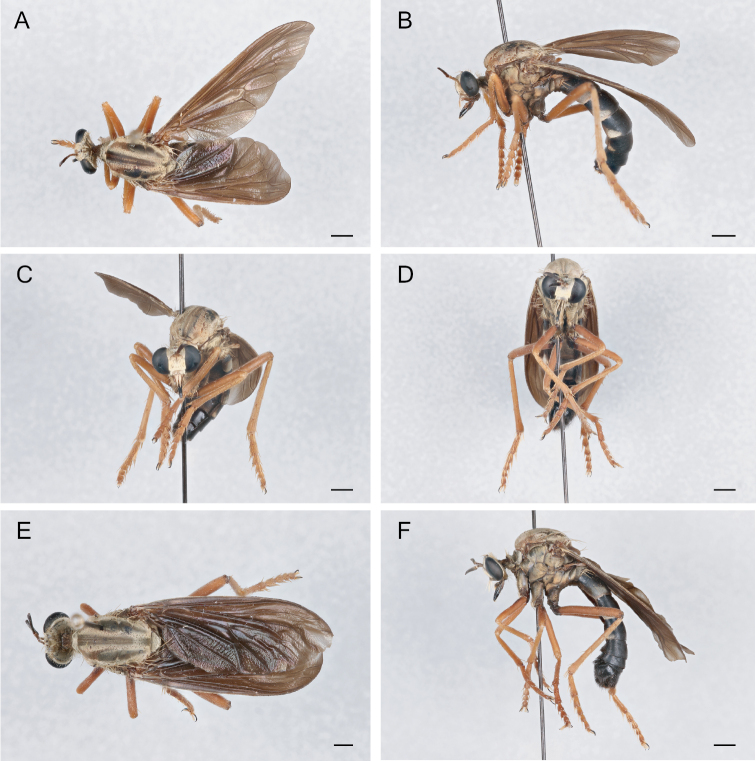
*Saropogonpritchardi* Bromley, 1934 Female (UCBMEP0280596): **A** dorsal view **B** lateral view **C** anterior view; Male (UCBMEP0280595): **D** anterior view **E** dorsal view **F** lateral view. Scale bars: 2 mm.

#### Distribution.

USA: New Mexico, Oklahoma, Texas, SimpleMappr: https://www.simplemappr.net/map/16991.

#### Type material examined.

United States of America • 1 ♂, holotype; Texas, Mills County; 20 July 1931; R. H. Painter; SEMC; SEMC1603974 • 1 ♂, 1 ♀, metatype; Oklahoma, Cimarron County, Boise City; 36°43'N, 102°30'W; 1271 m; 10 Jul 1933; A. E. Pritchard; USNM; USNMENT01819137, USNMENT01819532.

#### Other material examined.

Suppl. material 1.

#### Comments.

The holotype is housed at SEMC and information about it can be found here: https://biodiversity.ku.edu/node/1095/.

### ﻿ Saropogon purus

Taxon classificationAnimaliaDipteraAsilidae

Curran, 1930

CC48757D-781F-54F3-8C02-6F74C1544059

**Figure 21.**
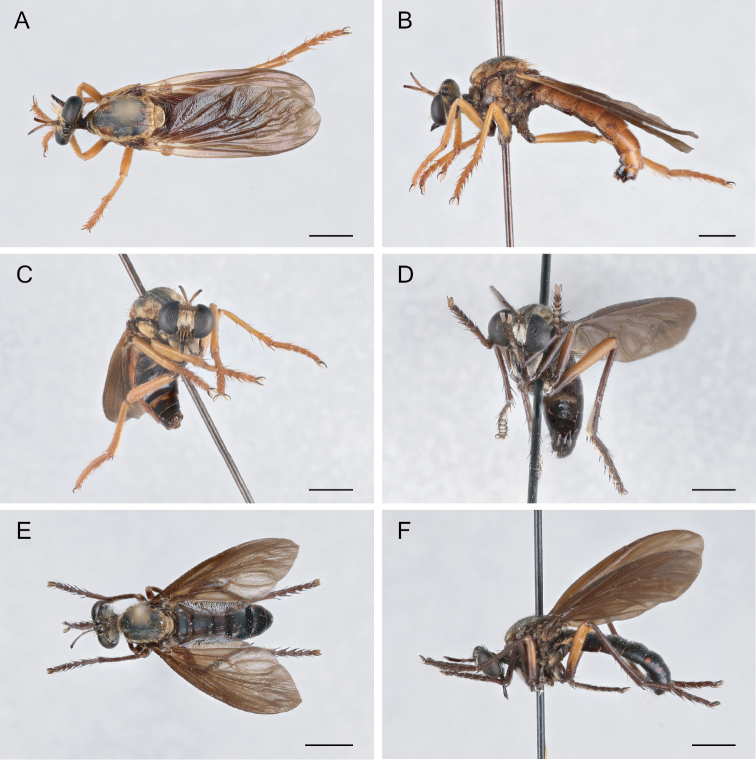
*Saropogonpurus* Curran, 1930 Female (UCBMEP0280564): **A** dorsal view **B** lateral view **C** anterior view; Male (USNMENT01830082): **D** anterior view **E** dorsal view **F** lateral view. Scale bars: 2 mm.

Figs 4E, F , 5M, N , 21 , 26 , 33

Saropogon purus Curran, 1930: 3.

#### References.

Curran 1930 (key and original description); Curran 1931: 2 (key); Martin and Wilcox 1965: 383 (catalog); Wilcox 1966: 129 (key); Fisher and Wilcox 1997: 4 (catalog).

#### Diagnosis.

The broad, brown wings easily distinguish this species from others (Fig. 5). It is a sexually dimorphic species (Fig. 4). Male abdomen and legs are black, metathoracic femora in part reddish; female abdomen and legs are mostly yellowish red, coxae densely deep with golden pubescence. Body length 11–13 mm; wing length 7–9 mm. Flight time July to August.

#### Distribution.

USA: Arizona; Mexico: Sinaloa, Sonora, SimpleMappr: https://www.simplemappr.net/map/16992.

#### Type material examined.

United States of America • 1 ♂, holotype; Arizona, Pima County, Kits Peak Rincon, Baboquivari Mts.; 31°57'N, 111°33'W; 1234 m; 1–4 August 1916; F. E. Lutz; AMNH • 1♀, allotype; same collection data as holotype; AMNH.

#### Arizona material examined.

United States of America • 1 ♂; Cochise County, Willcox; 32°15'N, 109°49'W; 1274 m; 13 July 1944; F. H. Parker; UAIC • 2 ♀; Gila County, Globe; 33°23'N, 110°47'W; 1074 m; 26 Jul 1987; Parker; USNM; USNMENT01819537, USNMENT01819572 • 1♀; same collection data as for preceding; 13 July 1956; F. H. Parker; UAIC • 1 ♂; same collection data as for preceding; 15 July 1943; F. H. Parker, UAIC • 1♀; same collection data as for preceding; 15 July 1948; F. H. Parker; UAIC • 1♀; same collection data as for preceding; 19 July 1947; F. H. Parker; UAIC • 1♀; same collection data as for preceding; 20 July 1956; F. H. Parker; UAIC • 2♀; same collection data as for preceding; 27 August 1955; F. H. Parker; UAIC • 1 ♂; same collection data as for preceding; 28 July 1952; F. H. Parker; UAIC • 1 ♂; Gila County, San Carlos; 33°20'N, 110°27'W; 809 m; 11 July 1936; F. H. Parker; UAIC • 1 ?; Maricopa County, 1.5 mi. NE of Desert Vista Point, Payson Highway; 33°40'N, 111°30'W; 753 m; 02 August 1969; R. Wielgus; ASUHIC; ASUHIC0139662 • 1 ?; Pima County, 2.1 mi. S. of Gibbon Mountain, Santa Catalina Mountains; 32°18'N, 110°44'W; 1006 m; 20 Aug. 1972; O. Francke, M. Kolner; ASUHIC0139664 • 1 ♂; Pima County, Baboquivari Mts.; 31°48'N, 111°36'W; 1234 m; 19 July 1950; J. G. Rosen; USNM; USNMENT01830301 • 1 ♂; Pima County, Baboquivari Mts.; 31°47'N, 111°34'W; 1776 m; USNM; USNMENT01819457 • 1♀; Pima County, Box Canyon Santa Rita Mountains; 33°08'N, 111°12'W; 592 m; 05 August 1978; D. S. Verity; USNM; USNMENT01830083 • 1♀; Pima County, Brown Canyon; 31°28'N, 110°17'W; 1219 m; 27 July 1973; E. M. Fisher; USNM; USNMENT01830285 • 1♀; same collection data as for preceding; 28 July 1983; Werner, Olson; UAIC • 1♀; Pima County, Espero Canyon 10 mi. NW of Tucson; 32°18'N, 110°49'W; 844 m; 10 August 1975; B. Page; UAIC • 1♀; Pima County, Snata Rita Exp. Range; 32°50'N, 110°51'W; 1120 m; 26 July, 1971; E. Yensen; UAIC • 1 ♂; Santa Cruz County, 3 mi. W. Pina Blanca; 31°24'N, 111°08'W; 1476 m; 07 July 1984; A. J.. Gilbert, R. A. Clark, J. C. Ball; USNM; USNMENT01830302 • 1 ♂; Santa Cruz County, Pena Blanca Area, Vic. Atascosa Trail; 31°24'N, 111°08'W; 1433 m; 05 July 1972; D. G. Marqua; USNM; USNMENT01830082 • 1 ?; Yavapai County, Cordes; 34°18'N, 112°10'W; 1150 m; 09 August 1971; M. Kolner; ASUHIC; ASUHIC0139663.

#### Other material examined.

Suppl. material 1.

#### Comments.

Most specimens have two scutellar bristles, but Wilcox (1966) noted that some have four.

### ﻿ Saropogon pyrodes sp. nov.

Taxon classificationAnimaliaDipteraAsilidae

57A35F0E-162E-5403-9EC4-DA86DC11A862

https://zoobank.org/3B057DFB-5B32-445D-AE22-037E7FD4C0C8

Figs 1 , 22 , 23 , 24 , 25 , 26 , 27 , 34

#### Diagnosis.

The species is distinguished from congeners by its deep red color, hyaline wings, gracile body, white pubescence on the posterior margin of T1–7, and T3 is typically darker than the other tergites (Fig. 1).

#### Description.

**Figure 22.**
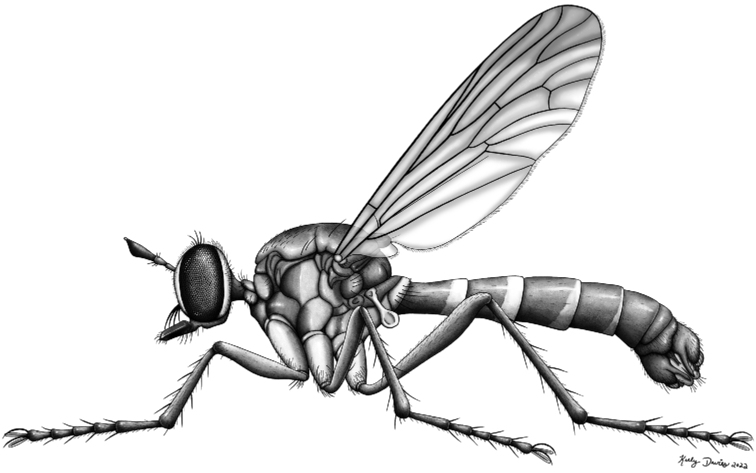
Habitus drawing of male *Saropogonpyrodes* sp. nov. by Keely Davies.

**Figure 23.**
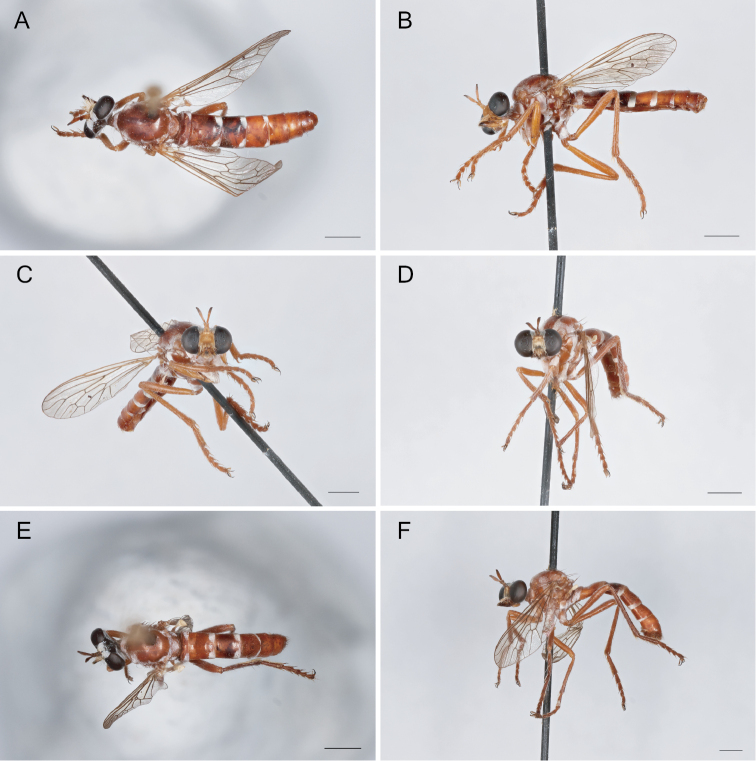
*Saropogonpyrodes* sp. nov. paratype female: **A** dorsal view **B** lateral view **C** anterior view; holotype male: **D** anterior view **E** dorsal view **F** lateral view. Scale bars: 2 mm.

**Male.** Holotype (Figs 22, 23D–F).

***Head*.** (Fig. 23) Wider than high; vertex slightly depressed (less than 60° angle on median margin of compound eye); facial swelling not developed and with gold pubescence; mystax 24 white macrosetae that are restricted to lower facial margin; ommatidia of different sizes, at least some median ommatidia distinctly larger; postgena with its posterior margin simple and smooth; frons with gray pubescence, white setose; ocellar tubercle with gray pubescence, with white setae and macrosetae; vertex with gray pubescence and white setae; median occiput sclerite with several white macrosetae; postocular setae slightly angled anteriorly distally, with white macrosetae; occiput predominately with gray pubescence and white setae; postocciput non-pubescent, with white and brown macrosetae.

***Proboscis and maxillary palpus*.** (Fig. 23) Proboscis straight, subequal in length to an eye when viewed from the front, pale brown to dark brown distally; postmentum with white setae ventrally; prementum with white setae proximo-ventrally; labella reduced, apex blunt; maxillary palpus pale brown to orange, with yellow setae and macrosetae, non-pubescent.

***Antenna*.** (Fig. 23) Pale brown to dark brown distally, with pale gray pubescence; scape approximately as long as pedicel, short white setae dorsally and long white macrosetae ventrally; pedicel white and pale brown setae distally; postpedicel tapering distally, medially broadest, short, approximately the same length as scape and pedicel combined, asetose; stylus composed of one element, asetose, with an apical seta-like sensory element in cavity of stylus.

***Thorax*.** (Fig. 23) Pale brown to orange, with white pubescence; proepisternum with gray pubescence, with white setae and macrosetae; cervical sclerite long, with white setae; antepronotum with white pubescence, with white setae and macrosetae; postpronotum with white pubescence, with white setae; postpronotal lobe setose; pleuron with white pubescence; proepimeron asetose; anepisternum asetose; anepisternum supero-posterior asetose; anterior basalare asetose, with white pubescence; posterior basalare asetose, with white pubescence; anepimeron asetose, anterior half with white pubescence, posterior half non-pubescent; katepisternum asetose, anterior half non-pubescent, posterior half with white pubescence; katepimeron asetose, non-pubescent; katergite with white setae and macrosetae, with white pubescence; meron and metanepisternum asetose, with white pubescence; metakatepisternum asetose, with white pubescence; metepimeron asetose, and with white pubescence; anatergite asetose, with white pubescence; scutum predominantly with gray pubescence; scutum brown with white setae and macrosetae; scutal setae with small sockets; two notopleural setae; one supraalar seta; one postalar seta; many (> 4) short white dorsocentral (dc) setae; many (> 4) short white acrostichal setae; many (> 4) short white medial setae on posterior scutum (between dc setae); scutellum with gray pubescence; discal scutellar setae absent; apical scutellar setae present, two long brown macrosetae.

***Leg.*** (Fig. 23) Pale brown to orange, non-pubescent, at least some setae dorso-ventrally flattened, others circular; coxae orange, with gray pubescence, with white setae and macrosetae; prothoracic femur flattened with white setae ventrally and long white setae dorsally; prothoracic tibia with short white setae except the antero-ventral surface has short gold setae, one or two yellow macroseta on distal end of ventral side, with white macrosetae: four in a postero-dorsal row, five short ones in a postero-ventral row, one or two long macrosetae in a postero-ventral row; prothoracic tibia with sigmoid spur, originating antero-ventrally directly from tibia; mesothoracic coxa with gray pubescence, with white setae and macrosetae; mesothoracic femur ventrally asetose except for two white macrosetae on proximal end, short white macrosetae sparsely covering the rest; mesothoracic tibia with short white setae, white macrosetae: three in an antero-dorsal row, 2 in 1 antero-ventral row, four in a dorsal row, three in a postero-ventral row; metathoracic coxa with gray pubescence, with white setae and macrosetae; metathoracic femur with long white setae and macrosetae; metathoracic tibia with white macrosetae: three in a antero-dorsal row, three in an antero-ventral row, three in a dorsal row, three in a postero-ventral row, straight; tarsus with proximal pro, mes, and met tarsomeres as long as following two tarsomeres combined, with brown macrosetae; pulvilli well-developed (as long as claw); claw smoothly arched distally, pointed; empodium setiform, and well developed (as long as pulvilli).

**Figure 24.**
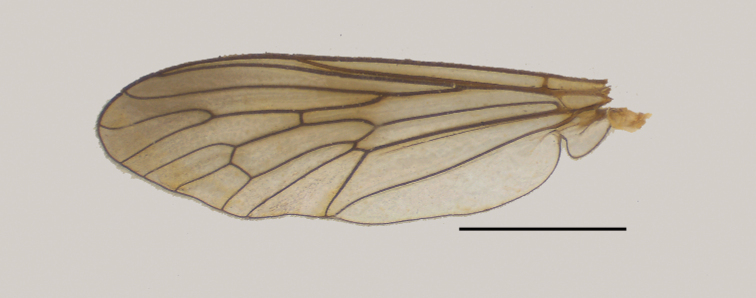
*Saropogonpyrodes* sp. nov. wing. Scale bar: 2 mm.

***Wing*.** (Fig. 24) 8 mm. Hyaline, without microtrichia; posterior wing margin with microtrichia arranged in a single plane.

**Figure 25.**
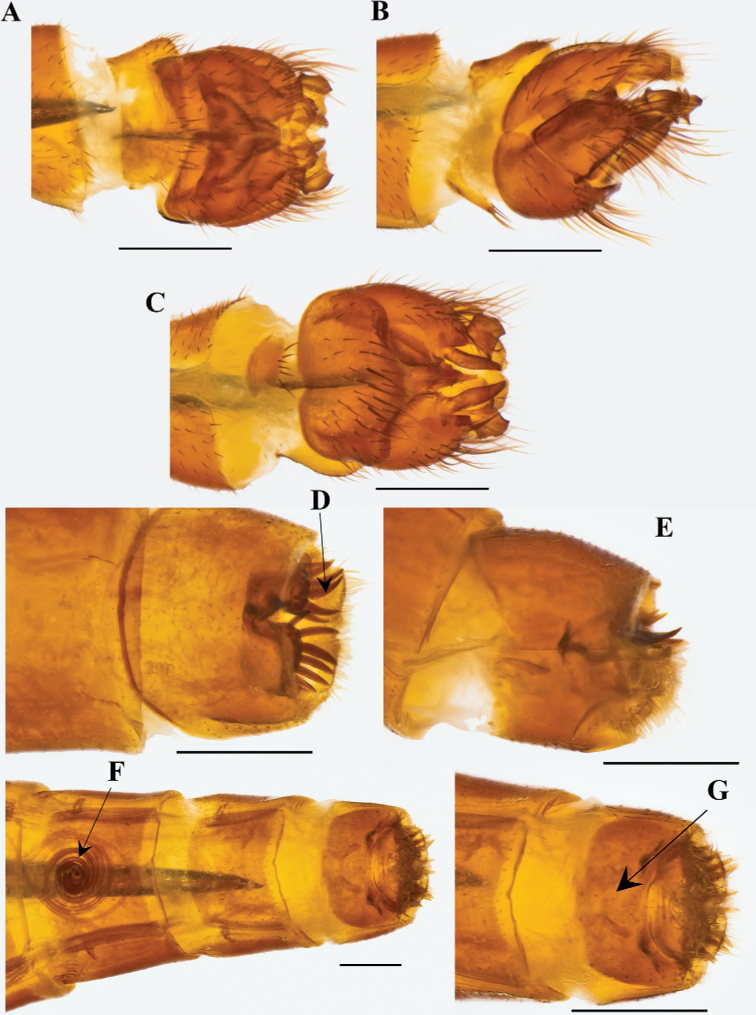
*Saropogonpyrodes* sp. nov. terminalia. Male (USNMENT01819155): **A** dorsal view 75× **B** lateral view 75× **C** ventral view 75×; female (UAIC1128818): **D** dorsal view 80×, arrow indicating acanthophorites (spines) **E** lateral view 95× **F** ventral view of T6–9 40×, arrow indicating spiral spermathecal reservoir **G** ventral view of T8–9 80×, arrow indicating “X” shaped furca. Scale bars: 1 mm.

***Abdomen*.** (Figs 6, 25) Pale brown to orange with some tergites brown dorsally; tergite sculpture smooth and setae with small sockets only; T1 white setose, laterally with long white macrosetae, predominantly with gray pubescence, medially non-pubescent, entirely sclerotized medially, dorsal surface smooth and without protuberances; T2–8 entirely sclerotized, white setose, setae short medially and longer laterally, predominantly pale brown to orange, predominantly non-pubescent with gray pubescent band on posterior margin, band thinner dorso-medially; T2–8 marginal and medial macrosetae absent; S1–8 brownish orange, with short white setae, and with pale gray pubescence.

***Male abdomen*.** (Fig. 25A–C) S8 simple, reduced rectangular sclerite; hypopygium rotated ~ 90° and pointing posteriorly; epandrium separated medially, joining proximally, and unfused; hypandrium well-developed and rectangular; hypandrium and epandrium approximating laterally, but not fused proximally; hypandrium and gonocoxites entirely free; gonocoxal apodeme present and short; gonostyli present and positioned distally on gonocoxites; cerci free and not fused medially; lateral ejaculatory process present and with a large cylindrical sclerite; one functional phallic prong; hypandrium with posterior margin simple with no distinct projections; sperm sac appearing weakly sclerotized; ejaculatory apodeme is a single plate.

***Female abdomen*.** (Fig. 25D–G) S7 and T7 are normally developed, without any modifications; segments eight and following comprising ovipositor; setae on T8 are directed anteriorly; T8 with anterior rectangular apodeme and entirely fused to T8; S8 plate-like with hypogynial valves extending; T9 and T10 partly fused; T10 divided into two heavily sclerotized acanthophorite plates with eight acanthophorite spurs on each plate; three equally large spermathecae, common spermathecal duct short, and not extending beyond tip of furca, individual spermathecal ducts long; spermathecal reservoir formed by coiled ducts and heavily sclerotized spermathecae contained within three most posterior segments; furca divided anteriorly into two lateral sclerites, H-shaped; furcal apodeme present, short and platelike.

***Length*.** Body length 10 mm; wing length: 6 mm.

#### Holotype condition.

The holotype is in good condition and is not missing any parts.

#### Type material.

United States of America • 1♂, holotype; Arizona, Pima County, 7 mi. N. Tucson; 33°47'N, 111°34'W; 740 m; 04 Sep. 1968; D. R. Miller, J. E. Lauck; USNM; USNMENT01199000 • 1♀, 7♂, paratypes; same data as for holotype; USNM; USNMENT01819173, USNMENT01199055, USNMENT01819150, USNMENT01819585, USNMENT01819580, USNMENT01819176, USNMENT01819472 • 3♂, paratypes; same data as for holotype; CASENT; USNMENT01819175, USNMENT01819179, USNMENT01819155 • 1♂, paratype; same data as for holotype; BMEC; USNMENT01819167 • 1♂, paratype; Arizona, Pima County, 4 mi. N. Continental; 31°54'N, 110°57'W; 844 m; 11 Aug. 1964; M. E. Irwin; USNM; USNMENT01819500 • 1♀, 1♂, paratypes; Arizona, Santa Cruz County, Juan Bautista De Anza Trail Amado; 31°44'N, 111°02'W; 916 m; 31 Aug. 2018; C. W. Melton; UAIC; UAIC1128818, UAIC1128819; BugGuide: https://bugguide.net/node/view/1588371, 1588372, 1588341, 1588340, 1588338 • 1♂, paratype; same data as for proceeding; TAM; USNMENT01819495.

#### Other material examined.

United States of America • 1♀; Arizona, Pima County, Green Valley; 31°50'N, 110°59'W; 943 m; 03 Sep 2016; K. Roragen; iNaturalist: https://www.inaturalist.org/observations/51920444 • 1♀; Arizona, Santa Cruz County, 0.7 km ExNE of Amado; 31°42'N, 111°03'W; 934 m; 05 Sep 2017; J. Gruber; BugGuide: https://bugguide.net/node/view/1439519; Flickr: https://www.flickr.com/photos/7432824@N07/albums/72157701454226641.

The holotype (1♂) and several paratypes (1♀ 7♂) of the new species have recently been deposited in USNM (as a donation from Eric Fisher); the rest of the paratypes will be split between BMEC (1♂), CASENT (3♂), UAIC (1♀ 1♂), TAM (1♂). Information and pictures of the holotype are available on the Smithsonian National Museum of Natural History Search the Department of Entomology Collections website: http://n2t.net/ark:/65665/36f568a66-098a-4932-8900-92113e4b58b9.

#### Distribution.

**Figure 26.**
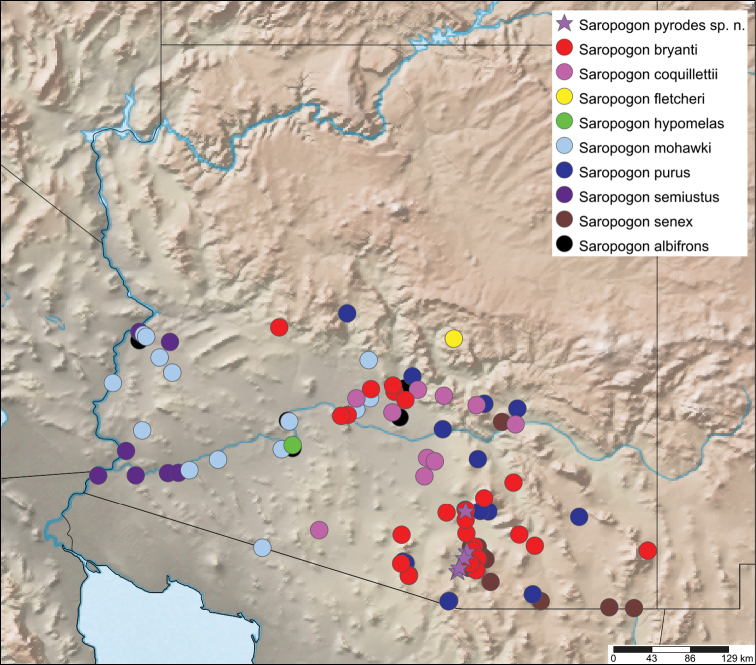
Focused map of the Arizona distribution of Nearctic *Saropogon* (Diptera: Asilidae). Map created with SimpleMappr on January 25, 2022, and available at: https://www.simplemappr.net/map/17143.

USA: Arizona (Fig. 26) https://www.simplemappr.net/map/17143.

#### Biology.

**Figure 27.**
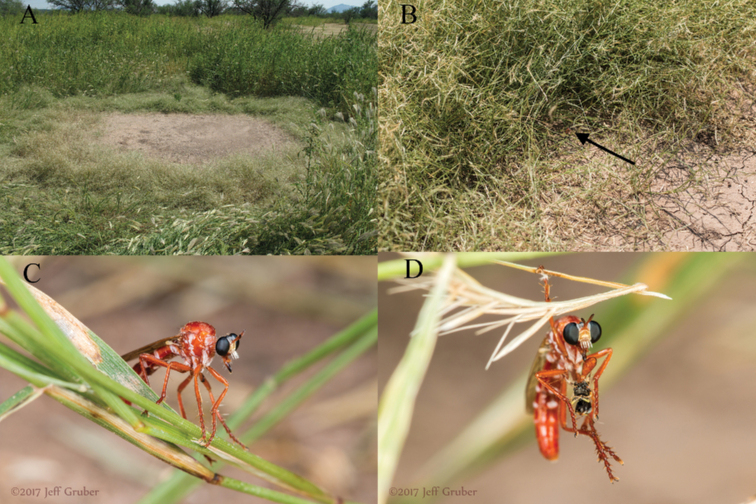
*Saropogonpyrodes* sp. nov. in natural habitat at ~ 0.7 km ENE of Amado in southern Arizona on September 5, 2017 **A** habitat overview **B** habitat detail with *S.pyrodes* included (arrow) **C** close-up of male perching **D** close-up of male consuming a bee (Hymenoptera: Apidae). Photographs by Jeff Gruber.

Jeff Gruber photographed specimens of *Saropogonpyrodes* sp. nov. and its habitat (Fig. 27A, B). *S.pyrodes* sp. nov. is seen here perching/hunting on a grass, most likely *Boutelouaaristidoides* (Poaceae; Fig. 27C), on the edge of a sandy clearing as well as consuming its prey (Fig. 27D) in the typical hanging position observed in other Dasypogoninae species.

Jeff Gruber described some behavior (Figs 1, 27) on Flickr: “Found this beauty as I was walking back to my car mid-afternoon on a very warm day. It was hanging around the low grasses at the periphery of a *Pogonomyrmex* ant nest in grassland type habitat on floodplain(?) of Santa Cruz River, which at the time was a dry wash. It alternated perches between the low grasses, short dead stems poking up from the soil, and the soil surface”. Original post: https://www.flickr.com/photos/7432824@N07/36417103883/in/faves-157063159@N04/.

#### Etymology.

Named for the fly’s bright, fiery red color: *pyrodes* is Greek for fire-like.

#### Comments.

In 1964, Mike Irwin collected the first record of this species, a male from four miles north of Continental, Arizona. He gave the specimen to Joseph Wilcox to identify. Then in 1968, Miller collected twelve specimens (11 ♂ and 1 ♀) from just north of Tucson, Arizona. He also donated this collection to J. Wilcox. The second author borrowed the specimens from Wilcox in approximately 1979 when he started a Ph.D. program at the University of California, Riverside. He considered describing this unique fly but never did. Finally, in 2017, beautiful photographs by Jeff Gruber (Fig. 27A–D) of this species appeared on BugGuide (https://bugguide.net/node/view/1439519), an online community where naturalists post and identify images of arthropods from the United States and Canada. Because of this, the second author immediately knew that this fly was long overdue for description, resulting in this manuscript.

*Saropogonbryanti* and *S.senex* have been collected within 10 km of the type locality of *S.pyrodes*. *Saropogonpurus* and *S.coquillettii* can also be found in the area; the material examined showed specimens within 60 km of *S.pyrodes* collection sites. *Saropogonhypomelas*, *S.fletcheri*, *S.albifrons*, and *S.mohawki* are all found within 200 km (Fig. 26). *Saropogonpyrodes* typically flies later in the season (Aug. – Sep.) than *S.bryanti* and *S.senex* (Jun. – Aug.), *S.purus* (Jul.), and *S.albifrons* (Apr. – Jun.). *Saropogoncoquillettii* (May – Sep.), *S.fletcheri* and *S.mohawki* (Jun – Oct.), and *S.hypomelas* (Jun. – Sep.) have longer flight seasons but are uncommon in the later months.

### ﻿ Saropogon semiustus

Taxon classificationAnimaliaDipteraAsilidae

Coquillett, 1904

C0EF0CFF-FF30-5C64-98D4-DFBA13FCBA99

Figs 26 , 28 , 31

Saropogon semiustus Coquillett, 1904: 186.

#### References.

Back 1909: 351 (key and redescription); Curran 1930: 2 (key); Curran 1931: 2 (key); Martin and Wilcox 1965: 383 (catalog); Wilcox 1966: 130 (key and comments); Fisher and Wilcox 1997: 4 (catalog).

**Figure 28.**
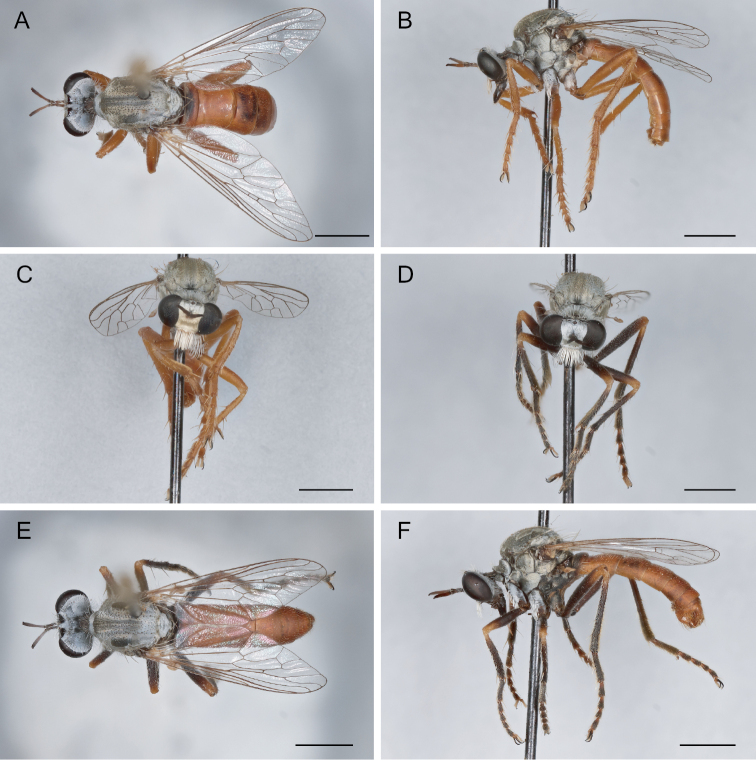
*Saropogonsemiustus* Coquillett, 1904 Female (USNMENT01830085): **A** dorsal view **B** lateral view **C** anterior view; Male (USNMENT01830084): **D** anterior view **E** dorsal view **F** lateral view. Scale bars: 2 mm.

#### Diagnosis.

This species most closely resembles *Saropogonhyalinus* and *S.albifrons* but can be easily separated by its smaller size and dense grayish pubescence on the face, thorax, scutellum, and coxae. Abdomen mostly polished with sides of T1 and a spot on the posterior corner of T2–5, with gray pubescence (sometimes absent in males). Legs in male black, except red at tips of femora; legs in female are reddish. Antennae are yellowish brown. Wings hyaline. Body length 8–10 mm; wing length 7–8 mm. Flight time April – June.

#### Distribution.

USA: Arizona, California; Mexico: Sonora, SimpleMappr: https://www.simplemappr.net/map/16994.

#### Type material examined.

United States of America • 1 ♂, holotype; California, San Diego County; 32°42'N, 117°09'W; 38 m; Coquillett; USNM; USNMENT01199020.

#### Arizona material examined.

United States of America • 1 ♂; La Paz County, Parker, Osborn Well Road, 1.6 km E. of Route 95, white sand dunes; 34°07'N, 114°15'W; 150 m; 02 May 2008; T. Dikow, E. Fisher; USNM; USNMENT00870563 • 1 ♂, 1 ♀; La Paz County, Cactus Plain Wilderness Study Area, off Swansea Road near aqueduct; 34°00'N, 113°57'W; 365 m; 27 April 2015; T. Dikow; USNM; USNMENT01115214, USNMENT01115055 • 4 ♂, 6 ♀; La Paz County, Parker, Osborn Well Road, 1.6 km E. Route 95; 34°07'N, 114°15'W; 150 m; 02 May 2008; T. Dikow, E. Fisher; USNM; USNMENT01830325, USNMENT01830326, USNMENT01830327, USNMENT01830328, USNMENT01830329, USNMENT01830330, USNMENT0183031, USNMENT01830332, USNMENT01830333, USNMENT01830334 • 1 ?; Yuma County, 1 mi. W. of Tacna; 32°42'N, 113°58'W; 102 m; 24 April 1966; J. H. Davidson, J. M. Davidson, M. A. Cazier; ASUHIC; ASUHIC0139671 • 1 ?; Yuma County, 19 mi. NE of Yuma; 32°55'N, 114°23'W; 128 m; 09 April 1966; J. H. Davidson, J. M. Davidson, M. A. Cazier; ASUHIC; ASUHIC0139669 • 1 ♂; Yuma County, 5 mi. E. Tacna; 32°42'N, 113°51'W; 104 m; 17 June 1965; F. D. Parker; BME; BMEP0280492 • 1 ♂; same collection data as for preceding; R. M. Bohart; BME; BMEP0280493 • 3 ?; Yuma County, 6 mi. SE. of Parker; 34°05'N, 114°12'W; 208 m; 23 April 1966; J. H. Davidson, J. M. Davidson, M. A. Cazier; ASUHIC; ASUHIC0139665, ASUHIC0139666, ASUHIC0139667 • 1 ?; same collection data as for preceding; 14 May 1966; J. H. Davidson, J. M. Davidson, M. A. Cazier; ASUHIC; ASUHIC0139668 • 1 ?; same collection data as for preceding; 07 May 1966; J. H. Davidson, J. M. Davidson, M. A. Cazier; ASUHIC; ASUHIC0139672 • 1 ?; Yuma County, Ligurta; 32°40'N, 114°17'W; 604 m; 08 April 1966; J. H. Davidson, J. M. Davidson, M. A. Cazier; ASUHIC; ASUHIC0139670 • 1 ♀; Yuma County, Welton; 32°40'N, 114°40'W; 76 m; F. H. Parker; USNM; USNMENT01819552.

#### Other material examined.

Suppl. material 1.

#### Comments.

Photographs of the holotype can be found here: http://n2t.net/ark:/65665/3648f2ac9-3f50-4efb-9719-6f3128085846.

### ﻿ Saropogon senex

Taxon classificationAnimaliaDipteraAsilidae

Osten Sacken, 1887

B6201334-4F05-5097-A9C6-7FD4296F0C4F

**Figure 29.**
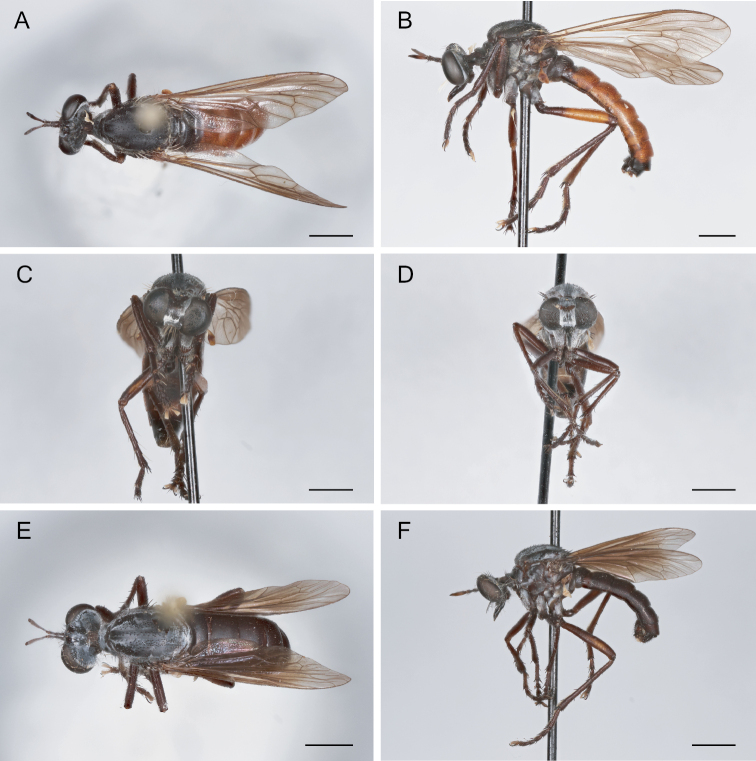
*Saropogonsenex* Osten Sacken, 1887 Female (UCBMEP0280483): **A** dorsal view **B** lateral view **C** anterior view; Male (UCBMEP0280489): **D** anterior view **E** dorsal view **F** lateral view. Scale bars: 2 mm.

Figs 4G, H , 5O–P , 26 , 29 , 34

Saropogon senex Osten Sacken, 1887: 179.
Saropogon aridus Curran, 1930: 3, junior synonym.

#### References.

Curran 1930: 2 (key, as *S.aridus*); Curran 1931: 2 (key, as *S.aridus*); Martin and Wilcox 1965: 383 (catalog); Wilcox 1966: 128 (key); Fisher and Wilcox 1997: 4 (catalog).

#### Diagnosis.

This species is mainly black with the hind femora of the female and sometimes of the male, reddish. Discal scutellar setae absent; four short apical scutellar macrosetae; scutum, anepisternum, and scutellum with grayish pubescence. Body length 10–12 mm; wing length 7–9 mm. Flight time June – August.

#### Distribution.

USA: Arizona; Mexico: Sinaloa, Sonora, Nayarit, SimpleMappr: https://www.simplemappr.net/map/16995.

#### Type material examined.

**Mexico** • 1 ♂, holotype; Presidio; 29°33'N, 104°22'W; Forrer; NHMUK; NHMUK013933278; Record 1427186.

#### Arizona material examined.

United States of America • 1 ?; Cochise County, 1 mi. E. of Douglas; 31°20'N, 109°31'W; 1241 m; 26 Jul. 1962; M. A. Cazier; ASUHIC; ASUHIC0139680 • 1 ♀; Cochise County, 8920 Hereford S Bryerly Ct.; 31°24'N, 110°13'W; 1500 m; 24 June 2016; N. E. Woodley; USNM; USNMENT01819474 • 1 ♂; same collection data as for preceding; 25 June 2016; N. E. Woodley; USNM; USNMENT01819469 • 1 ♂, 1 ♀; same collection data as for preceding; 27 June 2017; N. E. Woodley; USNM; USNMENT01819464, USNMENT01819484 • 1 ♀; same collection data as for preceding; 10 July 2017; N. E. Woodley; USNM; USNMENT01819454 • 1 ♀; same collection data as for preceding; 14 July 2017; N. E. Woodley; USNM; USNMENT01819459 • 1 ♀; same collection data as for preceding; 09 July 2019; N. E. Woodley; USNM; USNMENT01819479 • 1 ♀; Cochise County, San Bernardino Ranch; 31°20'N, 109°16'W; 1143 m; August; F. H. Snow; USNM; USNMENT01819159 • 1 ♂; Cochise County, Texas Pass Dragon Mts; 31°59'N, 105°02'W; 1107 m; 21 July 1984; J. C. Burne; UAIC .• 2 ♀; Gila County, Globe; 33°23'N, 110°47'W; 1074 m; 03 August 1949; F. H. Parker; USNM; USNMENT01819174, USNMENT01819527 • 1 ♀; same collection data as for preceding; 27 July 1956; F. H. Parker; UAIC • 1 ♂, 1 ♀; same collection data as for preceding; 1076 m; 07 August 1970; F. H. Parker; UAIC • 2 ♀; Gila County, Hayes Mt.; 33°12'N, 110°36'W; 1517 m; 25 August, 1957; F. H. Parker; UAIC • 1 ♀; Gila County, San Carlos; 33°20'N, 110°27'W; 806 m; 29 July, 1967; F. H. Parker; UAIC • 1 ♂; Pima County, 10 mi. E. Continental; 31°51'N, 110°48'W; 1264 m; 18 July 1961; Werner, Nutting; UAIC • 1 ♂; Pima County, 10 mi. SE. Sahuarita; 31°50'N, 110°51'W; 914 m; 21 July 1977; Olson, Hetz; UAIC • 1 ♂, 1 ♀; Pima County, 3 mi. E. Sahuarita; 31°57'N, 110°55'W; 843 m; 31 July 1963; V. L. Vesterby; BME; BMEP0280477, BMEP0280478 • 1 ?; Pima County, 4 mi. N. of Madera Canyon; 31°44'N, 110°56'W; 1086 m; 25 July 1966; J. M. Davidson, M. A. Cazier; ASUHIC; ASUHIC139683 • 1 ?; Pima County 8 mi. N. of Santa Rita Exp. Sta.; 31°56'N, 110°51'W; 905 m; 17 July 1970; M. Kolner, S. Szerlip; ASUHIC; ASUHIC139684 • 2 ?; Pima County, 8 mi. NW of Santa Rita Exp. Sta.; 31°47'N, 110°57'W; 949 m; 17 July 1970; M. Kolner, S.. Szerlip; ASUHIC; ASUHIC139686, ASUHIC139687 • 1 ♂; Pima County, Brown Canyon, Baboquivari Mts; 31°28'N, 110°17'W; 1527 m; 28 July 1983; Werner, Olson; UAIC • 1 ♀; Pima County, Santa Rita Mts.; 31°49'N, 110°46'W; 1813 m; 01 August 1941; R. H. Beamer; BME; BMEP0280476 • 1 ♀; same collection data as for preceding; R. H. Beamer, C. H. Martin; BME; BMEP0280472 • 1 ♀, 1 ?; same collection data as for preceding; 09 August 1930; T. F. Winburn, R. H. Painter; CASENT; CASENT8427344, CASENT8427345 • 1 ?; Pima County, Santa Rita Range Reserve; 31°43'N, 110°52'W; 1775 m; 15 July 1970; M. Cazier, J. Bigelow, L. Welch; ASUHIC; ASUHIC0139685 • 1 ♂; Pima County, Santa Rita Mts.; 31°49'N, 110°46'W; 1814 m; 31 June 1941; F. H. Parker; USNM; USNMENT01199040 • 1 ♂; same collection data as for preceding; 31 July 1944; F. H. Parker; USNM; USNMENT01199009 • 1 ♂; Pima County, Tucson, vic. Ina/Oracle; 32°19'N, 110°58'W; 770 m; 23 July 1988; W. L. Nutting; UAIC • 1 ♀; Pima or Santa Cruz County, Santa Rita RR; 31°35'N, 110°43'W; 1308 m; 15 August 1953; F. H. Parker; USNM; USNMENT01819139 • 1 ♂; Santa Cruz County, Santa Rita Mts. Madera Canyon; 31°44'N, 110°56'W; 1086 m; 15 July 1972; D. G. Marqua; USNM:USNMENT01830378 • 1 ♀; same collection data as for preceding; 24 July 1976; D. G. Marqua; USNM; USNMENT01830379 • 4 ♂, 3 ♀; same collection data as for preceding; 07–09 August 1962; E. M. Fisher; USNM; USNMENT01830365, USNMENT01830366, USNMENT01830367, USNMENT01830368, USNMENT01830369, USNMENT01830370, USNMENT01830371 • 1 ♂; same collection data as for preceding; 12–14 July 1961; E. M. Fisher; USNM; USNMENT01830372 • 2 ?; same collection data as for preceding; 25 July 1966; J. M. Davidson, M. A. Cazier; ASUHIC; ASUHIC0139681, ASUHIC0139682 • 1 ?; same collection data as for preceding; 26 August 1964; R. H. Crandall; LACM; LACMENT579126 • 2 ?; same collection data as for preceding; 01–06 August 1965; R. H. Crandall; LACM; LACMENT579128, LACMENT579129 • 1 ?; same collection data as for preceding; 06 August 1965; R. H. Crandall; LACM; LACMENT579127 • 2 ♂, 2♀; same collection data as for preceding; 13 July, 1958; R. M. Bohart, USNM, USNMENT01830374, USNMENT01830375, USNMENT01830376 • 2 ♂, 7♀; same collection data as for preceding; 31 July 1958; R. M. Bohart; BME; BMEP0280479, BMEP0280480, BMEP0280481, BMEP0280482, BMEP0280483, BMEP0280484, BMEP0280485, BMEP0280486; USNM; USNMENT01830373 • 1 ♀; same collection data as for preceding; 28 July 1979; S. Mannweiler; USNM; USNMENT01830377 • 1 ♀; same collection data as for preceding; 01 August 1960; S.. L. Wood, J. B. Karren, H. Shurtleff; BYU; BYUC215820 • 1 ?; Yavapai County, Badger Spring exit, 3.5 mi. NNE of Bumble Bee; 34°15'N, 112°06'W; 975 m; 04 August 1973; O. Francke, M. Kolner; ASUHIC; ASUHIC0139688.

#### Other material examined.

Suppl. material 1.

#### Comments.

Information about the holotype can be found here: https://data.nhm.ac.uk/record/bb909597-dedf-427d-8c04-4c02b3a24db3/1427186/1656374400000. At time of publication, there were no publicly available photographs of the specimen; however, pictures are scheduled to be posted to this link in the near future.

### ﻿ Saropogon solus

Taxon classificationAnimaliaDipteraAsilidae

Bromley, 1951

5EBD48EA-7F6D-56F0-93BA-531A672B3EF6

Figs 30 , 31

Saropogon solus Bromley, 1951: 15.

#### References.

Martin and Wilcox 1965: 383 (catalog); Wilcox, 1966: 128 (key); Fisher and Wilcox 1997: 4 (catalog).

**Figure 30.**
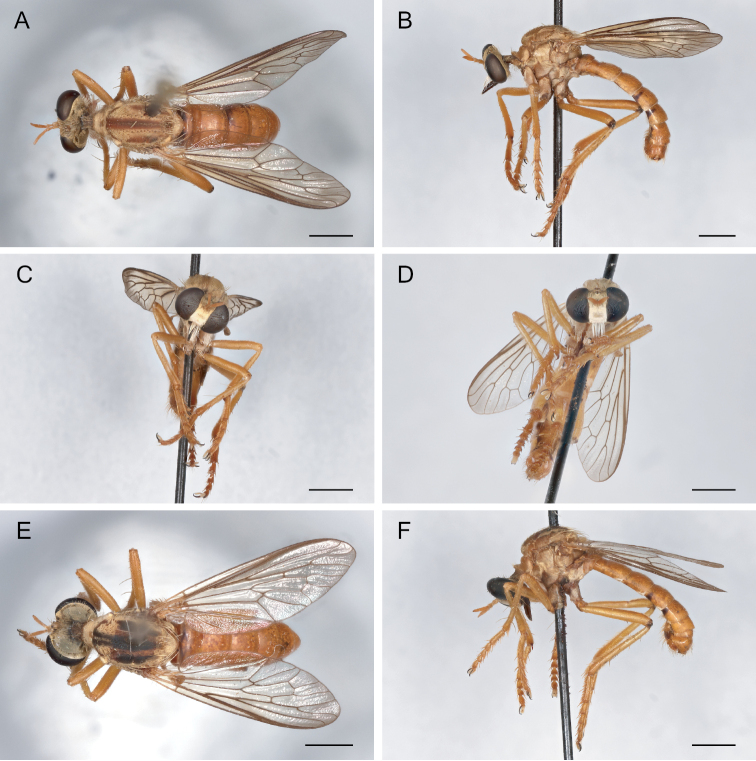
*Saropogonsolus* Bromley, 1951 Female (USNMENT01819178): **A** dorsal view **B** lateral view **C** anterior view; Male (USNMENT01819132): **D** anterior view **E** dorsal view **F** lateral view. Scale bars 2 mm.

#### Diagnosis.

This species is distinguishable from all other North American species by its lack of apical scutellar bristles. Wings are yellow tinged with gray tips; legs are reddish yellow. Body length 12 mm; wing length 8 mm. Flight time June – Aug.

**Figure 31.**
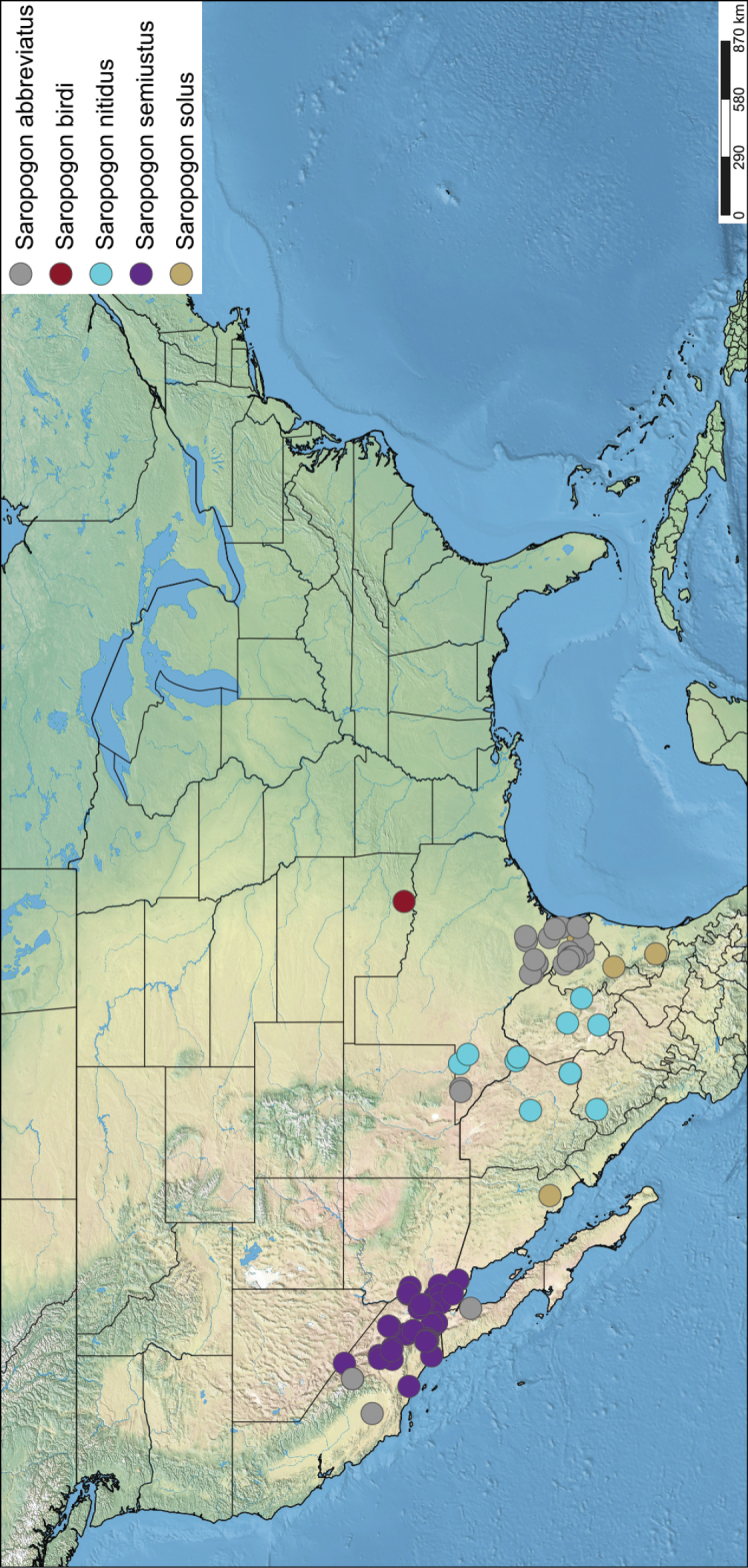
Distribution of *Saropogon* (Diptera: Asilidae) specimens studied for *S.abbreviatus*, *S.birdi*, *S.nitidus*, *S.semiustus*, and *S.solus*. Map created with SimpleMappr on July 25, 2022, and available at: https://www.simplemappr.net/map/18363.

#### Distribution.

USA: Texas; Mexico: Tamaulipas, SimpleMappr: https://www.simplemappr.net/map/16996.

#### Type material examined.

United States of America • 1 ♂, holotype; Texas, Hildago County; 26°27'N, 98°13'W; 39 m; 16 Jun 1933; S. W. Bromley; USNM; USNMENT01199013.

#### Other material examined.

Suppl. material 1.

#### Comments.

Photographs of the holotype are available at; http://n2t.net/ark:/65665/320c061d2-3a39-4baf-9836-909bdf168a64.

**Figure 32.**
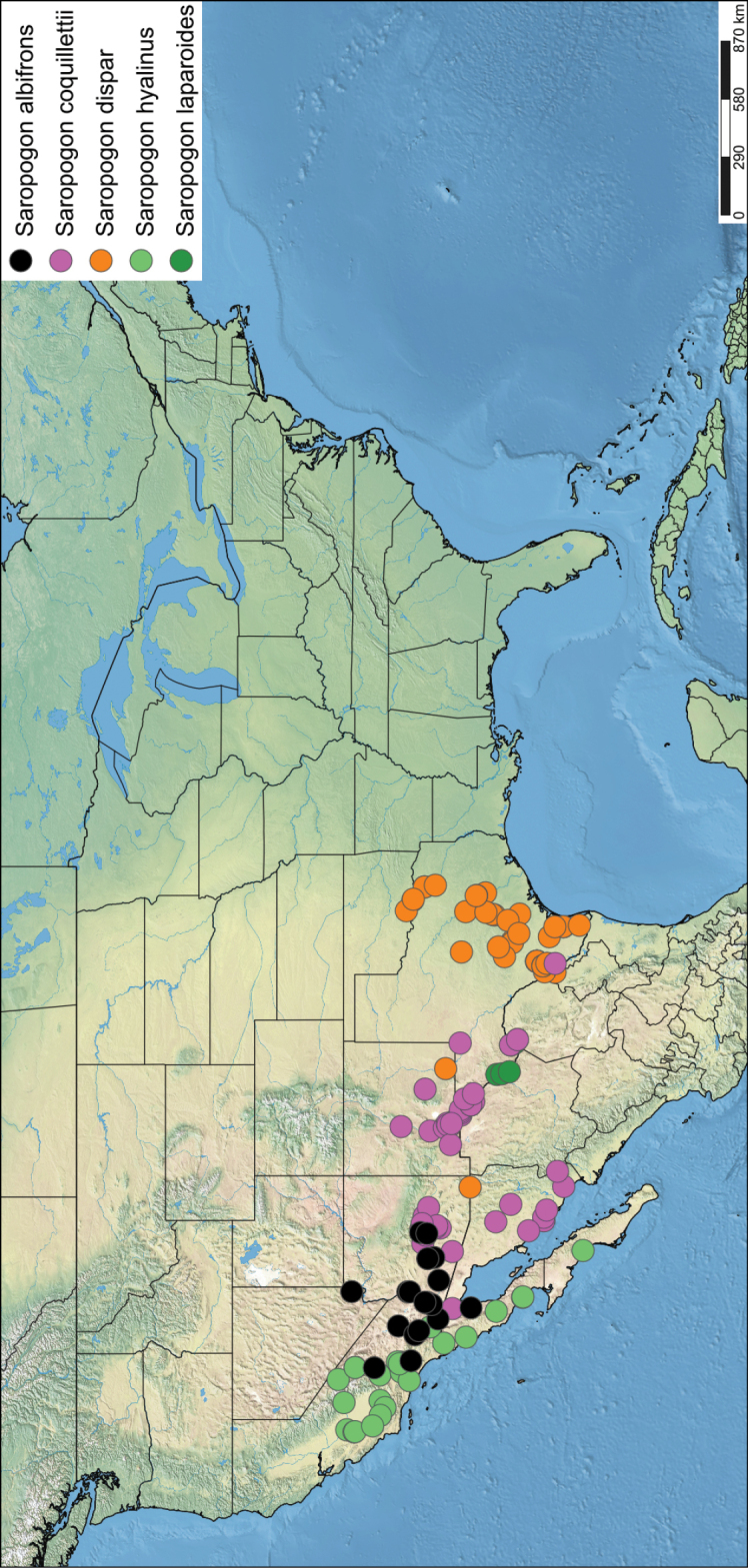
Distribution of *Saropogon* (Diptera: Asilidae) specimens studied for *S.albifrons*, *S.coquillettii*, *S.dispar*, *S.hyalinus*, and *S.laparoides*. Map created with SimpleMappr on July 25, 2022, and available at: https://www.simplemappr.net/map/18317.

## ﻿Discussion

The description of the unique species *Saropogonpyrodes* sp. nov., with the summary of our knowledge of the Nearctic *Saropogon* north of Mexico in the present study is an initial contribution to understanding the diversity of this genus. A future, more detailed revision of all Nearctic species including those occurring in Mexico, would be a natural extension of this project. Multiple new species from Sinaloa, Sonora, Durango, and Jalisco have been accumulating in the collection of the second author (recently donated to the USNM). Combined with specimens housed in Mexican natural history collections, these will provide the foundation for a comprehensive revision of the entire Nearctic fauna. With the description of *Saropogonpyrodes* sp. nov. there are now 20 species known from the USA, and *Saropogon* is now the third most speciose genus of Dasypogoninae after *Cophura* Osten Sacken (~ 34 spp.) and *Diogmites* Loew with (~ 25 spp.) in the Nearctic north of Mexico (see Fisher and Wilcox 1997). In terms of the entire Asilidae fauna of the Nearctic, *Saropogon* is the 14^th^ most species-rich genus (Fisher and Wilcox 1997; Geller-Grimm 2004).

**Figure 33.**
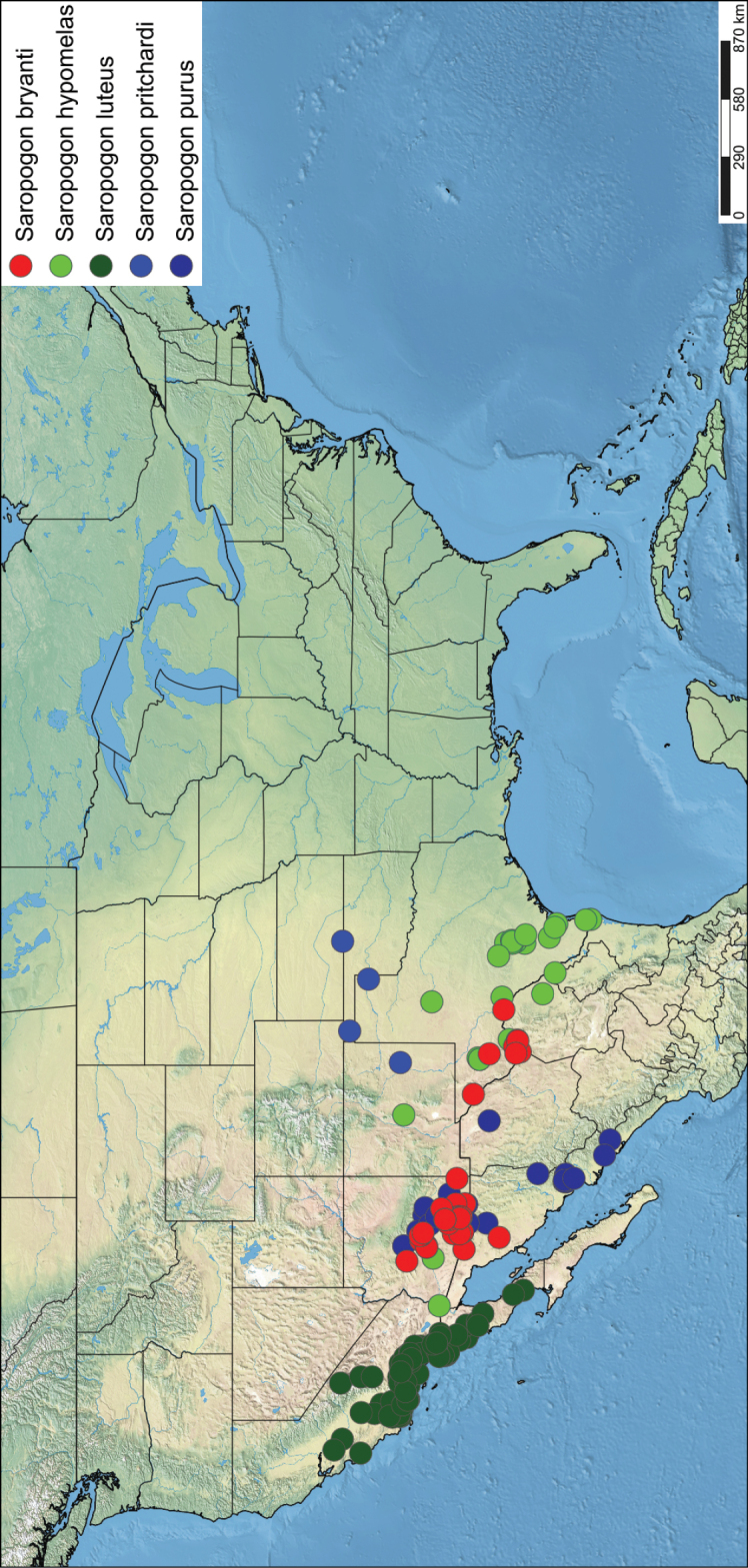
Distribution of *Saropogon* (Diptera: Asilidae) specimens studied for *S.bryanti*, *S.hypomelas*, *S.luteus*, *S.pritchardi*, and *S.purus*. Map created with SimpleMappr on July 25, 2022, and available at: https://www.simplemappr.net/map/18318.

There are a few morphological characters not previously mentioned that may prove useful for future species diagnosis and delimitation. The most apparent are the pubescence patterns on the dorso-median occiput (part or all of the median occipital sclerite). Of the species examined, *Saropogonalbifrons*, *S.bryanti*, *S.coquillettii*, and *S.dispar* have minimal to no patterning with solid pubescence. *Saropogonhyalinus*, *S.luteus*, *S.mohawki*, *S.nitidus*, *S.purus*, *S.semiustus*, *S.senex*, and *S.pyrodes* sp. nov. have two non-pubescent spots directly adjacent to slightly posterior to, the ocellar tubercle. Particularly distinct patterns occur in *Saropogonmohawki* where the cuticle showing through the two non-pubescent spots is pale brown instead of black as in the other species examined; *S.purus* has one large non-pubescent spot behind the ocellar tubercle, and *S.pyrodes* sp. nov. has two non-pubescent spots, but they appear much rounder and larger than in the other material examined. These are far from concrete descriptions, but it shows further observation may be warranted. Another character we would like to reexamine in future studies is the dependence on the number of apical scutellar setae in the identification of *Saropogon*. This character has been heavily relied upon in past identification keys despite it being known for being inconsistent within species. Our key attempts to replace this character with other more dependable characters and only rely on apical scutellar setae where necessary (e.g., *S.mohawki* and *S.hyalinus*).

**Figure 34.**
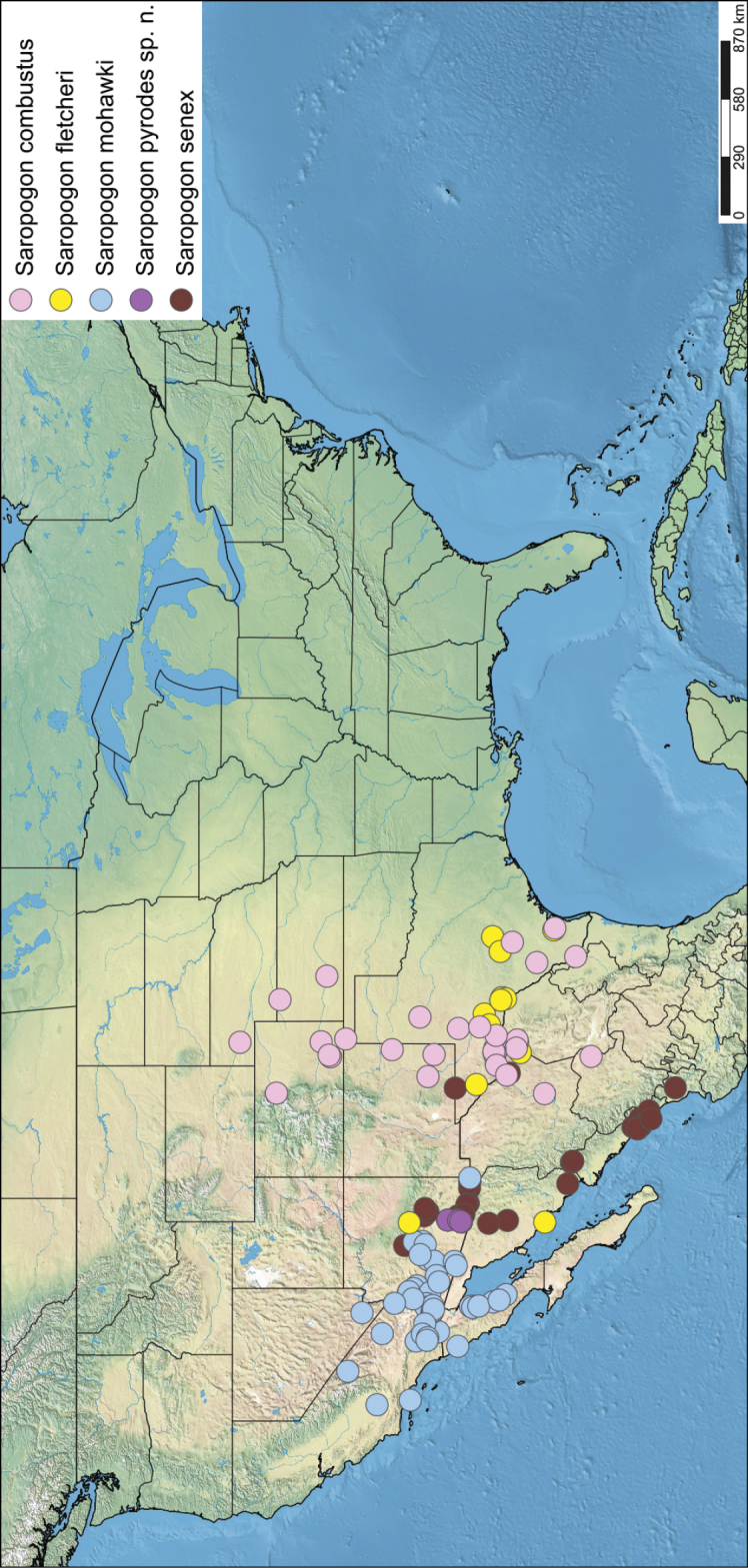
Distribution of *Saropogon* (Diptera: Asilidae) specimens studied for *S.combustus*, *S.fletcheri*, *S.mohawki*, *S.pyrodes* sp. nov., and *S.senex*. Map created with SimpleMappr on July 25, 2022, and available at: https://www.simplemappr.net/map/18362.

Platforms like iNaturalist and BugGuide have greatly facilitated communication between community and professional entomologists. *Saropogonpyrodes* sp. nov. is an excellent example of how community involvement can assist in the discovery and, ultimately, the description of new species. These community-based websites are a relatively new resource that scientists are learning to utilize in their research, and we hope to encourage future participation on both sides of the professional plane.

## Supplementary Material

XML Treatment for Saropogon

XML Treatment for Saropogon abbreviatus

XML Treatment for Saropogon albifrons

XML Treatment for Saropogon birdi

XML Treatment for Saropogon bryanti

XML Treatment for Saropogon combustus

XML Treatment for Saropogon coquillettii

XML Treatment for Saropogon dispar

XML Treatment for Saropogon fletcheri

XML Treatment for Saropogon hyalinus

XML Treatment for Saropogon hypomelas

XML Treatment for Saropogon laparoides

XML Treatment for Saropogon luteus

XML Treatment for Saropogon mohawki

XML Treatment for Saropogon nitidus

XML Treatment for Saropogon pritchardi

XML Treatment for Saropogon purus

XML Treatment for Saropogon pyrodes

XML Treatment for Saropogon semiustus

XML Treatment for Saropogon senex

XML Treatment for Saropogon solus

## Supplementary materials

Supplementary material 1 Locality information of all specimens examined Data type excel file Explanation note Locality information of all specimens examined. https://binary.pensoft.net/file/770878This dataset is made available under the Open Database License (http://opendatacommons.org/licenses/odbl/1.0/). The Open Database License (ODbL) is a license agreement intended to allow users to freely share, modify, and use this Dataset while maintaining this same freedom for others, provided that the original source and author(s) are credited.Charlotte H. E. Alberts, Eric M. Fisher

## References

[B1] Artigas JN Papavero N (1988) The American genera of Asilidae (Diptera): Keys for identification with an atlas of female spermathecae and other morphological details. II. Subfamily Dasypogoninae Macquart, with descriptions of new genera and species and new synonymies. Gayana. Zoología 52(3–4): 199–260. https://biostor.org/reference/100813

[B2] Back EA (1904) New species of North American Asilidae. Canadian Entomologist 36(10): 298–293. 10.4039/Ent36289-10

[B3] Back EA (1909) The Robber-flies of America, north of Mexico, belonging to the subfamilies Leptogastrinae and Dasypogoninae. Transactions of the American Entomological Society 35(1): 137–400. 10.5962/bhl.title.9381

[B4] Bellamy CL (2002) Coleoptera: Buprestoidea. In: Houston WWK (Ed.) Zoological Catalogue of Australia. CSIRO Publishing, Collingwood, Australia, 492 pp. [ISBN: 0643069003]

[B5] Bromley SW (1934) The Robber Flies of Texas (Diptera, Asilidae). Annals of the Entomological Society of America 27(1): 74–113. 10.1093/aesa/27.1.74

[B6] Bromley SW (1951) Asilid notes (Diptera), with descriptions of thirty-two new species. American Museum Novitates 1532: 1–36. https://hdl.handle.net/2246/2377

[B7] Cohen CM Noble K Cole TJ Brewer MS (2021) The phylogeny of robber flies (Asilidae) inferred from ultraconserved elements. Systematic Entomology 46(4): 1–15. 10.1111/syen.12490

[B8] Coquillett DW (1902) New Orthorrhaphous Diptera from Mexico and Texas. Journal of the New York Entomological Society 10: 136–141. https://www.jstor.org/stable/25002994

[B9] Coquillett DW (1904) New North American Diptera. Proceedings of the Entomological Society of Washington 6: 166–192. https://www.biodiversitylibrary.org/page/2345832

[B10] Coquillett DW (1910) The type-species of the North American genera of Diptera. Proceedings of the United States National Museum 37: 499–647. 10.5479/si.00963801.37-1719.499

[B11] Cumming JM Wood DM (2017) Adult morphology and terminology In: Brown BV, Borkent A, Cumming JM, Wood DM, Woodley NE, Zumbabo MA (Eds) Manual of Central American Diptera. Vol. 1. National Research Council Research Press, Ottawa, Ontario, 714 pp. [ISBN: 0660198339]

[B12] Curran CH (1930) New American Asilidae (Diptera). The American Museum of Natural History. American Museum Novitates 425: 1–21. http://hdl.handle.net/2246/2113

[B13] Curran CH (1931) New American Asilidae (Diptera). II. The American Museum of Natural History. American Museum Novitates 487: 1–25. https://hdl.handle.net/2246/3035

[B14] Dennis DS Lavigne RJ (1975) Comparative behavior of Wyoming robber flies II (Diptera: Asilidae). University of Wyoming Agricultural Experiment Station 30, 68 pp.

[B15] Dikow T (2009a) Phylogeny of Asilidae inferred from morphological characters of imagines (Insects: Diptera: Brachycera: Asiloidea). Bulletin of the American Museum of Natural History 319: 1–175. 10.1206/603.1

[B16] Dikow T (2009b) A phylogenetic hypothesis for Asilidae based on a total evidence analysis of morphological and DNA sequence data (Insecta: Diptera: Brachycera: Asiloidea). Organisms, Diversity & Evolution 9(3): 165–188. 10.1016/j.ode.2009.02.004

[B17] Dikow T (2018) Asiloid flies: deciphering their diversity and evolutionary history. Asilidae generic classification *sensu* Dikow 2009. [Available from:] https://asiloidflies.si.edu/ [Accessed Oct 2, 2021]

[B18] Fisher EM Wilcox J (1997) Catalog of the robber flies (Diptera: Asilidae) of the Nearctic region. [distributed as unpublished document to Asilidae workers, widely used in community; new version underway with Cunnings, R. – to be published]

[B19] GBIF Secretariat (2021) *Saropogon* Loew, 1847. GBIF Backbone Taxonomy. [Checklist dataset available from:] https://www.GBIF.org [Accessed 4 October 2021]

[B20] GBIF.org (2022) GBIF Registry of Scientific Collections. [Available from:] https://www.gbif.org/grscicoll

[B21] Geller-Grimm F (2004) A world catalogue of the genera of the family Asilidae (Diptera). Studia Dipterologica 10(2): 473–526.

[B22] Google Earth Pro (2021) Version 7.3.4.8248. [Available through:] https://www.google.com/earth/versions/ [Accessed 3 October 2021]

[B23] Hardy GH (1926) A new classification of Australian robberflies belonging to the subfamily Dasypogoninae (Diptera, Asilidae). Proceedings of the Linnean Society of New South Wales 51: 305–312. https://biostor.org/reference/105137

[B24] Hull FM (1956) Some Asilidae (Diptera). Entomological News 67: 131–135. 10.1080/00222935608655832

[B25] Hull FM (1962) Robber flies of the world: the genera of the family Asilidae. In: Smithsonian Institution, United States National Museum. Bulletin 224. Part 2. Smithsonian Institution, United States National Museum, Washington, DC, 431–906. 10.5479/si.03629236.224

[B26] Hurd PD (1952) Revision of the Nearctic species of the pompilid genus *Pepsis* (Hymenoptera: Pompilidae). Bulletin of the American Museum of Natural History 98: 257–334. http://hdl.handlenet/2246/1025

[B27] Hurd PD Linsley EG (1975) Some insects other than bees associated with *Larreatridentata* in the southwestern United States. Proceedings of the Entomological Society of Washington 77: 100–120. [ISSN: 0013-8797]

[B28] Johnson CW (1903) A new genus and four new species of Asilidae. Psyche 10(323): 111–114. 10.1155/1903/25137

[B29] Lavigne RJ (2016) Predator-prey database. https://www.gellergrimm.de/catalog/lavigne.htm [accessed 10 July 2022]

[B30] Lehr PA (1988) Family Asilidae. In: Soos A, Papp L (Eds) Catalogue of Palaearctic Diptera, Budapest, 197–326. [ISBN: 9780444996022]

[B31] Linsley EG (1960) Ethology of some bee-and wasp killing robber flies of southeastern Arizona and Western New Mexico (Diptera: Asilidae). In: Linsley EG, Smith RF, Steinhaus EA, Usinger RL (Eds) University of California Publications in Entomology, University of California Press, Berkeley and Los Angeles 16(7): 357–392.

[B32] Loew H (1847) Ueber die europäischen Raubfliegen (Diptera Asilica). Linnaea Entomologica 2: 384–568. 10.5962/bhl.title.11475

[B33] Loew H (1874) Neue nordamerikanische Dasypogonina. Berliner Entomologische Zeitschrift 18(3–4): 353–377. 10.1002/mmnd.18740180322

[B34] Londt JGH (1980) Afrotropical Asilidae (Diptera) 4. The genus *Pegesimallus* Loew, 1858 (=Lagodias Loew, 1858; Neolaparus Williston, 1889), including species from other zoogeographical regions and the descriptions of the two new genera, *Brevirostrum* and *Caroncoma*. Annals of the Natal Museum 24: 233–347. https://hdl.handle.net/10520/AJA03040798_563

[B35] Londt JGH (1997) Afrotropical Asilidae (Diptera) 29. A review of the genus *Saropogon* Loew, 1847 (Dasypogoninae). Annals of the Natal Museum 38: 137–157. https://hdl.handle.net/10520/AJA03040798_167

[B36] Londt JGH Dikow T (2017) Chapter 48. Asilidae (assassin flies or robber flies). In: Kirk-Spriggs AH Sinclaire BJ (Eds) Manual of Afrotropical Diptera Volume 2.Nematocerous Diptera and lower Brachycera. Suricata 5. Pretoria: South African National Biodiversity Institute, 1097–1182. [ISBN: 9781828224129]

[B37] Martin CH Papavero N (1970) A catalogue of the diptera of the Americas south of the United States 35b Family Asilidae. Museu de Zoologia, Universidade de São Paulo, 35b.1–35b.139. 10.5962/bhl.title.110114

[B38] Martin CH Wilcox J (1965) Family Asilidae. In: Stone A Sabrosky CW Wirth WW Foote RH Coulson JR (Eds) A catalog of the diptera of America north of Mexico.U. S. Government Printing Office, Washington D.C., 360–1116. https://handle.nal.usda.gov/10113/CAT87208336

[B39] Mesaglio T Soh A Kurniawidjaja S Sexton C (2021) First known photographs of living specimens’: The power of iNaturalist for recording rare tropical butterflies. Journal of Insect Conservation 25(5–6): 905–911. 10.1007/s10841-021-00350-7

[B40] Nichols (1989) Torre-Bueno glossary of entomology. New York Entomological Society, New York, USA, 840 pp. [ISBN: 0913424137]

[B41] Osten Sacken CR (1887) Fam. Asilidae. Biologia Centrali-Americana 1: 167–213. 10.5962/bhl.title.730

[B42] Papavero N (1973) Studies of Asilidae (Diptera) systematics and evolution. I. A preliminary classification in subfamilies. Arquivos de Zoologia 23(3): 217–274. 10.11606/issn.2176-7793.v23i3p217-274

[B43] Pollock DA (2021) Robber flies (Diptera: Asilidae) as predators of harvester ant workers (Hymenoptera: Formicidae: *Pogonomyrmex*) in Eastern New Mexico and West Texas. The Southwestern Naturalist 65(1): 19–27. 10.1894/0038-4909-65.1.3

[B44] Sakhvon VV (2020) Review of the genus *Saropogon* Loew, 1847 (Diptera: Asilidae) from Russia, Transcaucasia and central Asia, with description of three new species. Zootaxa 4860(4): 577–591. 10.11646/zootaxa.4860.4.7 33055883

[B45] Shorthouse DP (2010) SimpleMappr, an online tool to produce publication-quality point maps. [Available from:] https://www.simplemappr.net [Accessed 24 November 2020]

[B46] Sweetman H (1958) The principles of biological control. W. C. Brown, Dubuque, 560 pp.

[B47] Thorp RW (1973) Prey records for robber flies of the Lake Texoma region Oklahoma (Diptera: Asilidae). The Pan-Pacific Entomologist 49(1): 89–90.

[B48] VanDyk J [Ed.] (2021) BugGuide.Net: Identification, images, and information for insects, spiders and their kin for the United States and Canada. Iowa State University. [Available from:] https://bugguide.net/ [Accessed 4 October 2021]

[B49] Wilcox J (1936) Asilidae, new and otherwise, from the south-west, with a key to the genus *Stichopogon*. The Pan-Pacific Entomologist 13(1–2): 37–45. https://www.biodiversitylibrary.org/page/53411556

[B50] Wilcox J (1966) New species and a key to the species of *Saropogon* Loew (Diptera: Asilidae). The Pan-Pacific Entomologist 42(2): 127–136. https://www.biodiversitylibrary.org/page/53621647

[B51] Winterton SL Guek HP Brooks SJ (2012) A charismatic new species of green lacewing discovered in Malaysia (Neuroptera, Chrysopidae): The confluence of citizen scientist, online image database and cybertaxonomy. ZooKeys 214: 1–11. 10.3897/zookeys.214.3220 PMC3426877 22936863

